# Ultrasound-sensitive microrobotic sensor with robust anchoring for long-term digestive lesion assessment

**DOI:** 10.1093/nsr/nwag179

**Published:** 2026-03-20

**Authors:** Chen Xin, Yuqiong Wang, Yihang Jiang, Bo Hao, Neng Xia, Jiaqi Zhu, Lin Su, Jinsheng Zhao, Xin Wang, Haojin Yang, Xianfeng Xia, Kai Fung Chan, Qingsong Xu, Dong Wu, Philip Wai Yan Chiu, Joseph Jao Yiu Sung, Li Zhang

**Affiliations:** Institute of Humanoid Robots, Department of Precision Machinery and Precision Instrumentation, University of Science and Technology of China, Hefei 230026, China; Department of Mechanical and Automation Engineering, The Chinese University of Hong Kong, Hong Kong 999077, China; Department of Mechanical and Automation Engineering, The Chinese University of Hong Kong, Hong Kong 999077, China; Department of Mechanical and Automation Engineering, The Chinese University of Hong Kong, Hong Kong 999077, China; Department of Mechanical and Automation Engineering, The Chinese University of Hong Kong, Hong Kong 999077, China; Department of Mechanical and Automation Engineering, The Chinese University of Hong Kong, Hong Kong 999077, China; Department of Mechanical and Automation Engineering, The Chinese University of Hong Kong, Hong Kong 999077, China; Department of Mechanical and Automation Engineering, The Chinese University of Hong Kong, Hong Kong 999077, China; Department of Mechanical and Automation Engineering, The Chinese University of Hong Kong, Hong Kong 999077, China; Department of Mechanical and Automation Engineering, The Chinese University of Hong Kong, Hong Kong 999077, China; Department of Mechanical and Automation Engineering, The Chinese University of Hong Kong, Hong Kong 999077, China; Department of Surgery, The Chinese University of Hong Kong, Hong Kong 999077, China; Chow Yuk Ho Technology Center for Innovative Medicine, The Chinese University of Hong Kong, Hong Kong 999077, China; Department of Electromechanical Engineering, Faculty of Science and Technology, University of Macau, Macau 999078, China; Institute of Humanoid Robots, Department of Precision Machinery and Precision Instrumentation, University of Science and Technology of China, Hefei 230026, China; Department of Surgery, The Chinese University of Hong Kong, Hong Kong 999077, China; Chow Yuk Ho Technology Center for Innovative Medicine, The Chinese University of Hong Kong, Hong Kong 999077, China; Lee Kong Chian School of Medicine, Nanyang Technological University, Singapore 308232, Singapore; Department of Mechanical and Automation Engineering, The Chinese University of Hong Kong, Hong Kong 999077, China; Department of Surgery, The Chinese University of Hong Kong, Hong Kong 999077, China; Chow Yuk Ho Technology Center for Innovative Medicine, The Chinese University of Hong Kong, Hong Kong 999077, China

**Keywords:** microrobot, magnetic actuation, robotic sensor, ultrasound imaging

## Abstract

Microrobots have achieved notable progress in targeted drug delivery for treating various diseases. However, employing the microrobot itself as a microsensor for remote and long-term lesion assessment remains challenging. Here, we design a bioinspired ultrasound-sensitive microrobotic sensor (USMS) capable of robust anchoring for wireless and long-term digestive disease treatment and assessment. The USMS integrates dual magnetic and focused ultrasound actuation for precise locomotion and on-demand drug delivery, while a bioinspired microneedle design secures stable anchoring against strong fluid flow (402 mm/s) and peristaltic forces (66.5 mN). More importantly, the air cavity design of USMS enables strong ultrasound reflection for remote and long-term sensing of protruding lesion radius to assess therapeutic efficacy. Overall, USMS demonstrates reliable on-demand drug delivery and lesion size sensing capabilities in mouse models. The USMS has the potential to serve as a versatile robotic platform and sensing system for monitoring lesion evolution in clinical disease management.

## Teaser

An ultrasound-sensitive microrobotic sensor with strong anchoring enables targeted drug delivery and long-term monitoring of digestive diseases.

## INTRODUCTION

To date, thanks to the development of micro-manufacturing technologies and smart materials [1–11], microrobots have become increasingly miniaturized [12–14], integrated [13,15], and intelligent [16–20]. These advancements have allowed microrobots to show great potential in areas such as targeted drug delivery [21–23] and disease treatment [24,25]. Among them, magnetic-responsive robots offer the advantages of programmable shape-morphing [26–28], wireless control [29–31], and high localization capabilities [32–34], making them ideal for targeted drug delivery. In the complex environment of the digestive tract, magnetic robots can achieve precise positioning and motion control, enabling targeted and sustained drug release for enhanced therapeutic outcomes [22,35]. However, using microrobots designed for targeted treatment as microsensors for long-term sensing of disease sites within the body remains a significant challenge.

Current approaches for sensing digestive lesions, such as computed tomography (CT) and X-ray imaging, are often expensive, invasive, and unsuitable for continuous sensing. Ultrasound imaging has gained increasing attention owing to its ease of use, absence of radiation, and capacity to visualize tissues at depths exceeding 10 cm with high spatial resolution [36–38]. However, direct sensing of deep digestive tract lesions remains challenging because of the low acoustic reflection contrast between lesions and surrounding tissues. Microbubble contrast agents have recently been employed to enhance ultrasound imaging resolution [39]. By amplifying ultrasound signal reflections, these agents markedly improve image contrast and sensitivity [40–42]. Therefore, the development of microrobots integrated with ultrasound-sensitive microbubbles holds great promise for achieving real-time tracking and positioning, while opening new avenues for remote and long-term lesion assessment *in vivo*.

**Figure 1.**
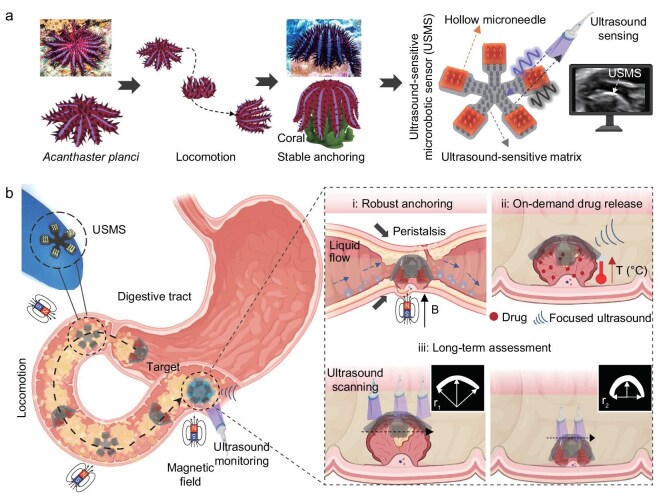
(a) Inspired by *Acanthaster planci* anchoring coral and defending from predators through soft arms full of multiple spines, we design ultrasound-sensitive microrobotic sensor (USMS) consisting of hollow microneedles and a magnetic ultrasound-sensitive matrix for robust anchoring, on-demand drug release, and long-term therapeutic assessment. Part of (a) licensed from Adobe Stock. (b) Schematic diagram of USMS with precise locomotion, robust anchoring, and long-term tumor size sensing in the digestive tract. The USMS is composed of hollow microneedles (MNs) and a magnetized soft body filled with air cavities of high ultrasound reflection. Due to shape morphing and strong acoustic reflection, the USMS could achieve robust anchoring, on-demand drug delivery, and long-term therapeutic efficacy sensing in the digestive tract.

Inspired by *Acanthaster planci* with shape morphing, precise locomotion, wrapping of prey, and defensive penetration in dynamic liquid environments, we design an ultrasound-sensitive microrobotic sensor (USMS) consisting of a programmable magnetized body with air cavities and thorn-like microneedle arrays, which exhibits strong ultrasound reflection, reversible shape morphing, and robust anchoring (Fig. 1a). The USMS can achieve on-demand drug release in the digestive tract via magnetic-controlled locomotion and focused ultrasound triggering (FUT). In addition, shape morphing and penetration enhance the USMS’s anchoring ability on complex curved surfaces against strong liquid flow and intestinal peristalsis. Based on powerful ultrasound reflection capability, USMS enables long-term sensing of tumor size to evaluate therapeutic effect (Fig. 1b). As a concept of application validation, on-demand drug delivery, robust anchoring, and long-term tumor size assessment using USMS are demonstrated in animal models. Overall, we develop a USMS-based platform with active targeting, morphing, anchoring, and ultrasound-sensing capabilities, promising practical applications in on-demand drug delivery and long-term disease assessment.

## RESULTS

### Fabrication and characterization of bioinspired USMSs

**Figure 2.**
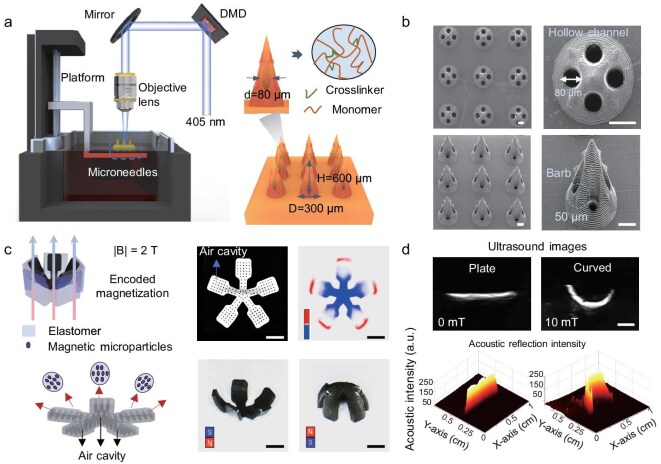
Fabrication and characterization of magnetic USMSs. (a) Microneedle arrays with multiple hollow channels and a cargo storage tank are fabricated by a μSLA system. DMD represents the digital microscope device. Each patch has nine hollow microneedles with a diameter of 300 μm and a height of 600 μm. (b) The scanning electron microscopy (SEM) images of hollow microneedles with channels of 80 μm diameter and barbs of 50 μm length. (c) The magnetization of the magnetic soft substrate is completed under a uniform magnetic field strength of 2 T with the assistance of a mold. The air-cavities for ultrasound sensing are presented by micro-CT. The magnetic flux density (B) profiles of the structure after magnetization are measured by the magneto-optical sensor. The substrate can switch the shape between wrapping and unwrapping under the opposite uniform magnetic field. (d) The air cavity-filled USMS on tissue models exhibits strong acoustic reflection under ultrasound imaging. Scale bars: (b) 100 μm, (c, d) 2 mm.

To achieve robust anchoring and on-demand drug release in deep tissue, we develop a magnetic USMS consisting of ultrasound-triggered hollow microneedles (MNs) loaded with chemotherapeutic drugs and a soft magnetic body doped with ferromagnetic microparticles (NdFeB MPs), where five arms integrate 45 microneedles (Fig. S1 and Table S1). As shown in Fig. 2a and Fig. S2, MNs with multiple hollow channels and a cargo storage tank are fabricated by micro-stereolithography (μSLA) technology [43]. The system setup mainly consists of a 3D printing platform, a focusing lens, and a digital micromirror device (DMD), which enables different light patterns at each layer to stack the target 3D structures [44]. The hollow MNs are based on a photopolymerizable material consisting of poly (ethylene glycol) diacrylate (PEGDA), polyvinylpyrrolidone (PVP), and dipentaerythritol hexaacrylate, capable of degradation in an alkaline environment (Fig. S3) [45]. Here, each hollow MN is designed with a diameter of 300 μm and a height of 600 μm. To facilitate drug storage and release, the MNs have a storage tank and four hollow channels with a diameter of 80 μm. In addition, there are eight barb structures (regular tetrahedrons of 50 μm length) on the surface of the MN to prevent it from slipping out after penetration. The scanning electron microscope (SEM) images in Fig. 2b show the morphology of the patch with nine MNs, where the tip diameter can reach 7 μm, as shown in the magnified SEM (Fig. S4). To manipulate the hollow MNs controllably by magnetic fields, we fabricate magnetic soft silicone substrates based on the molding technique (Fig. S5). The silicone elastomer (Ecoflex 00–30) and NdFeB MPs (5 μm) are mixed and then poured into the fabricated mold and cured at 50°C for 1 h. Subsequently, the magnetic soft substrate is programmed to be magnetized by placing it in a pre-prepared 3D mold under a magnetic field strength of 2 T (Fig. 2c). The magnetic substrate is incubated in artificial intestinal fluid, and over the 15-day evaluation period, no measurable weight loss or elemental release is detected, indicating preserved structural integrity and satisfactory biocompatibility (Figs S6 and S7). The higher the mass fraction of NdFeB MPs, the higher the magnetization strength exhibited by the magnetized soft substrates (Fig. S8). In addition, the soft substrate’s size is highly customizable (4–9 mm). Meanwhile, the magnetization radius of the soft substrate can be programmed to meet different sizes of the targets (Fig. S9). As shown in the magneto-optical image, the magnetization direction of the magnetic substrate is encoded at different locations. In this way, the magnetic patch can be switched between wrapping and opening under a uniform magnetic field of opposite directions (Fig. 2c). Finally, we choose a degradable polymer (poly(lactic-co-glycolic acid), PLGA) to connect the above two units [46]. Eventually, the intact USMS could perform wrapping actions at magnetic field strengths ranging from 0 to 30 mT, like an *A. planci*. In addition, bubbles are commonly used in medical ultrasound imaging contrast agents with strong acoustic reflection intensity. USMS is designed with an array of through-holes to embed bubbles for enhanced ultrasound imaging and finally for treatment sites sensing. As shown in Fig. 2d, USMS embedded in tissue-like hydrogels exhibits high ultrasound imaging resolution, which bends noticeably when the magnetic field is applied. Compared to tissue-like hydrogels, USMSs filled with microbubbles have stronger acoustic reflection intensity, which helps them to be identified in tissues for long-term sensing. Micro-CT observations provide direct three-dimensional evidence that the air-cavity architecture remains intact over 14 days (Fig. S10). Therefore, the air-cavity architecture maintains stable acoustic performance over 14 days under physiological conditions (Fig. S11).

### Mechanical, shape-morphing, and penetration tests of USMS

**Figure 3.**
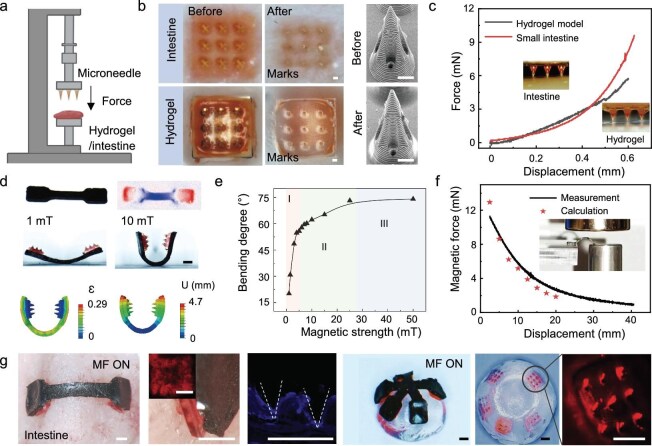
Mechanical, wrapping, and penetration tests of microneedles. (a) Set-up of the mechanical test system. (b) Bottom view of microneedle penetrating hydrogel model and isolated porcine small intestine. The tip of MN is not destroyed during penetration. (c) Relationship between penetrating force and the displacement of MNs. (d) A USMS with two patches is actuated under different magnetic field strengths (1–10 mT) to realize shape morphing. (e) Relationship between the bending angle of the USMS and the magnetic field strength (I, rapid increase, II, slow increase, and III, stable). (f) The mechanical force generated by USMS under different magnetic strengths. The five-pointed star represents the calculated value. (g) Penetration characterizations of the small intestine and semi-spherical hydrogel models by USMS. MF represents the magnetic field. Scale bars: (b) 100 μm, (d, g) 1 mm.

To guarantee that MNs can be pierced into the tissue under the actuation of a magnetic field, we have carried out a systematic mechanical analysis. In our work, we validate the mechanical penetration capability of microneedles on *ex vivo* porcine small intestinal tissues and tissue-like hydrogel models. The hydrogel model has viscoelasticity (hysteresis ∼0.54), fracture toughness (∼0.52 MPa), and Young’s modulus (∼11.16 kPa) comparable to the small intestine (hysteresis ∼0.53, fracture toughness ∼0.51 MPa, Young’s modulus ∼20.92 kPa) (Fig. S12). In addition, the porosity and hydration level of the hydrogel models are 42.67% and 84.09%, respectively (Fig. S13). The measurement of the mechanical penetration of the MNs is completed under compression using the set-up composed of precision mechanical sensors, nano-precision mobile platforms, and two imaging cameras (side view and bottom view). We can observe the microneedle penetrating the intestinal tissue and the hydrogel model through time-lapse images (Fig. 3a). Figure 3b shows the penetration process of intestinal tissue and the hydrogel model by MN (Movie S1). In our work, it is found that force per MN patch (<8 mN) can completely penetrate the isolated small intestine and hydrogel models (Fig. 3c). Compared with digestive tract tissues (10–100 kPa) [47,48] or hydrogel models, MN has a high compressive performance sufficient to complete the penetration. In addition to demonstrating that MNs have sufficient mechanical strength, we also characterize the deformability of the USMS driven by the magnetic field and the mechanical forces generated in the process.

Figure 3d shows that the deformation degree of USMS increases with the growth of magnetic field strength. To better observe and measure the degree of USMS deformation, we take a USMS with two patches as an example. When the magnetic field strength increases from 0 to 25 mT, the deformation angle of one side of the USMS increases from 0° to 70° (Fig. 3e). The corresponding simulation results show that the elastic body does not produce obvious stress concentration during deformation. When the magnetic field strength is up to 50 mT, the mechanical force generated by USMS can reach ∼9 mN (Fig. 3f, Tables S2 and S3, and Fig. S14), which is enough to penetrate intestinal tissue and hydrogel models. According to the calculation of the magnetic field force loaded on USMS, the remaining penetration force of USMS gradually increases when the radius of the wrapped object increases (Fig. S15). Therefore, we can reasonably design the size of the USMS according to the size of the manipulated object to provide suitable conformal and penetration effects. Compared to previous planar MNs, USMS can achieve shape morphing to adapt to many complex surfaces under the actuation of the magnetic field. Due to their high biocompatibility, degradability, and similar modulus to living tissue, Gelatin methacrylamide (GelMA) hydrogels are widely used as cell scaffolds for mechanical tests [49]. Thus, we print hydrogel models with various shapes to verify USMS’s superior wrapping and penetration ability. Side-view images of Fig. S16 show the MN penetrating the hydrogel model after the magnetic field is turned on, where the hydrogel model deformation and microneedle penetration can be observed (Movie S2). Furthermore, magnetic USMS enables the penetration of the isolated porcine small intestine and liver, and the successful penetration is further demonstrated by the MN traces in the frozen section images of the penetrated tissue. The red fluorescence image in Fig. 3g demonstrates that the fluorescent marks in the MN hollow channel diffuse uniformly through the small intestinal tissue. Ultimately, the USMS with five patches is developed for curved tissue model penetration and cargo delivery. In addition to the relatively regular tissue model, we have constructed various complex surfaces for USMS penetration, such as spherical, discoidal, segmental, and derivative tissue models (Fig. S17). After the USMS penetration, each model surface is left with obvious penetration marks.

### Controllable locomotion, wrap-around penetration, and on-demand drug delivery of USMS

**Figure 4.**
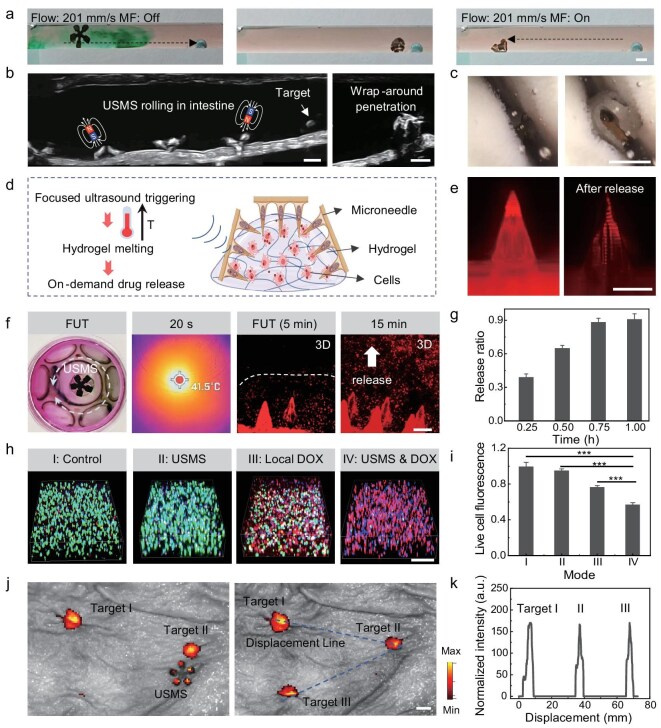
On-demand drug delivery of USMS by ultrasound triggering. (a) The USMS performs upstream locomotion under flowing fluid at a velocity of 201 mm/s. (b) Demonstration of the USMS locomotion and penetration in the small intestine under US imaging. (c) The medical endoscopy reveals that the USMS is capable of controlled locomotion within collapsed and mucus-filled small intestinal tissue. (d) Schematic diagram of USMS penetrating the tissue model and on-demand DOX molecules delivery by ultrasound triggering. FUT heats the hydrogel to its phase transition temperature, releasing DOX molecules. (e) Comparison before and after hollow MN releases DOX molecules. (f) Temperature of USMS under FUT within 20 s. Confocal microscopy images show DOX release and diffusion process in tissue model (FUT from 5 to 15 min). (g) DOX release rate from microneedles increases along with FUT time (0.25–1 h). Error bars represent the standard deviation (*n* = 3). (h) Comparison of therapy efficiency of different modes (I–IV: Control, USMS, Local DOX, and USMS & DOX), indicating enhanced efficiency of ultrasound-triggered drug release. (i) Fluorescence of live cells under different modes (I–IV) after 24 h. (j) Fluorescence images of multiple target positions after drug delivery, where the drug has no leakage during the movement of the USMS. (k) The drug fluorescence intensity plot under the robot’s motion trajectory demonstrates that the drug is only released on demand at the three targeted positions. Error bars represent the standard deviation (*n* = 4). ***P < 0.001. Scale bars: (a, b, c, j) 5 mm, (e, f, h) 300 μm.

The static magnetic field enables programmable deformation of the USMS, while the dynamic magnetic field can guide it to produce a variety of locomotion, such as crawling, swimming, rolling, and walking [50,51]. In our study, the USMS could not only roll on the substrate surface by applying a rotating uniform magnetic field strength (10 mT) but also walk toward the target position under an oscillating magnetic field (Fig. S18). In addition to dry substrates, USMS can also accomplish controlled movement on mucus-filled surfaces in an intestine phantom (Fig. S19) and an isolated porcine small intestine (Movie S3). Furthermore, USMS can overcome the fluid (∼201 mm/s) to complete controllable countercurrent movement (Fig. 4a). Although magnetic actuation is a wireless and precise locomotion control method [27,52,53], long-distance magnetically controlled motion is difficult and unsafe for the USMS. Based on enteric-coated capsules, USMS could be delivered non-invasively to the small intestinal lesion. Subsequently, guided by medical imaging, it is driven by a magnetic field and precisely targeted to the lesion. Here, fresh porcine tissues including the esophagus, stomach, and intestine are used to verify the feasibility of USMS targeted delivery *ex vivo* (Fig. S20). Guided by real-time ultrasound imaging, USMS can move flexibly in the intestine through magnetic actuation, including reaching the target area, wrapping, and penetrating the target tissue (Fig. 4b, Movie S4, part I). As shown in Fig. 4c, USMS can overcome mucus to achieve controllable movement in the collapsed intestine under endoscopic imaging. We also verified the compatibility of USMS with X-ray imaging, which has precise resolution and a large field of view (Fig. S21, Movie S4, section II).

The human digestive tract has always been full of folds and high-viscosity fluid, which makes it difficult for drug molecules to cross the mucus barrier to enter the inner tissue for treatment. In our work, USMS can wrap around curved lesions and break mucus barriers through microneedles for drug delivery in deep tissue. In addition, focus-ultrasound triggered drug release has high temporal and spatial precision. As shown in Fig. 4d, to evaluate the on-demand drug delivery by FUT, we fabricate a 3D tissue model (GelMA cultured with cells), which has been widely used to construct cell scaffolds and tissue models [49]. After focus ultrasound triggering, red fluorescence images show a significant release of hydrochloride doxorubicin (DOX) molecules loaded in MN (Fig. 4e). The drug molecule DOX is homogeneously mixed in a homemade hydrogel and loaded into the hollow channels of MNs. The hydrogel is a temperature-responsive biomaterial that converts from the solid phase to the liquid phase when the temperature is higher than 40°C [54,55]. When the USMS is triggered by ultrasound, the temperature of the localized area rises to reach the phase transition temperature of the hydrogel, which enables on-demand drug release in the deep tissue. In our study, the ultrasound source is generated by seven ceramic ultrasound transducers (1.7 MHz). They are embedded in a hemispherical holder that produces a focused acoustic field for heating at 5 cm from the center of the sphere (Fig. S22). When the tissue model is placed in the focused ultrasound zone, the temperature does not increase due to the low ultrasound absorption rate (Fig. S23). In contrast, USMS has higher ultrasound absorbance and can be heated from 29.1 to 41.5°C within 20 s for drug release (Fig. 4f and Fig. S24). The temperature during *in vivo* ultrasound actuation was limited to 40.3°C, remaining below the thermal damage threshold (Fig. S25). To make the ultrasound-triggered release of drugs applicable to a large animal model with thick tissue, an amplified focused ultrasound system consisting of 35 ultrasound transducers is developed (Fig. S26). The system can heat USMS across ∼10 cm of chicken breast tissue, demonstrating its feasibility for use in large animals. Subsequently, we verified the ultrasound-triggered release performance of the drug. Here, each microneedle patch could store 3.53–12.24 μg of drug molecules. The drug loading amount can be flexibly scaled up by the number of microneedles and drug concentration to meet the needs of large animals and adult humans (Fig. S27). As the release time increases, red fluorescence is delivered from the microneedle tip to the deeper part of the tissue model (Fig. 4f). Based on UV spectral analysis, DOX is released by ∼90% within 1 h (Fig. 4g and Fig. S28). As shown in Fig. 4h, the performances of various treatment modes are compared (modes I–IV: Control: without USMS & DOX delivery, USMS: USMS without DOX loading, Local DOX: local DOX delivery without USMS-mediated activation, and USMS & DOX: DOX-loaded USMS activated by ultrasound). Ultrasound triggering enables stronger intracellular drug delivery compared to drug release by diffusion, ultimately resulting in a significantly enhanced treatment. Statistical analysis of the treatment effect reveals that the fluorescence intensities of live cells decrease to 0.76 and 0.57 under mode III and mode IV, respectively (Fig. 4i). Moreover, multiple on-demand drug delivery can reduce the side effects of drug treatments compared to one-time drug delivery. Here, we validated multiple ultrasound-triggered treatments on a tissue model. The first ultrasound trigger can deliver a large amount of drug, covering 75% of the tissue model. In contrast, the 6th ultrasound trigger can still deliver the drug to ∼10% of the area (Fig. S29). Many gastrointestinal diseases, such as inflammatory bowel disease, colorectal cancer, and gastric ulcers, commonly present with multiple lesions distributed along different segments of the gastrointestinal (GI) tract [56–58]. Thanks to remote magnetic motion control and active drug delivery, our USMS can achieve targeted drug delivery to multiple lesions one by one. Fluorescent images show that the USMS accomplished drug release in three site-specific lesions. At the same time, the movement process avoids drug leakage leading to side effects (Fig. 4j). The intensity of drug release remained consistent across the three target regions (Fig. 4k), further validating the superiority of our system for targeted drug delivery in multiple specific lesions. More importantly, by combining magnetic field generation with image processing, USMS can autonomously move to multiple target locations with a high degree of reach accuracy (Fig. S30 and Movie S3).

### Robust anchoring and on-demand drug release of USMS in a rat model

**Figure 5.**
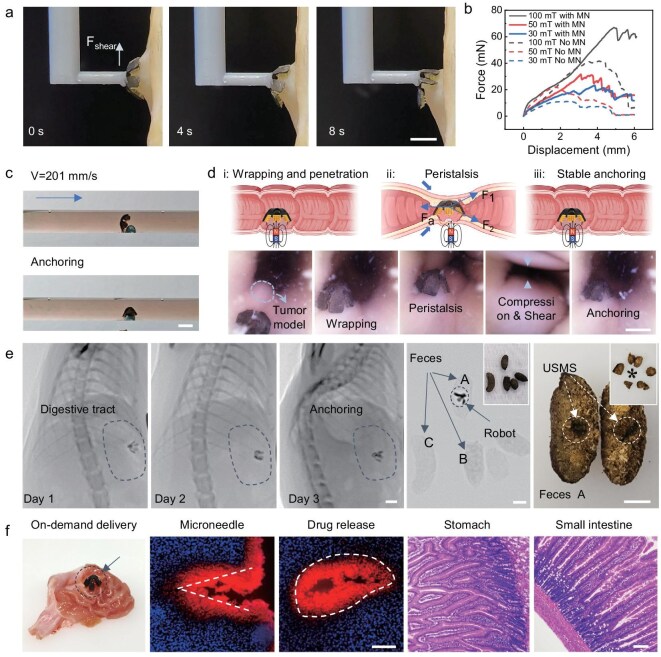
Robust anchoring and on-demand drug release of USMS *in vivo*. (a) Mechanical test of USMS anchoring tissues against shear forces (*F*_shear_). (b) The maximum shear force that USMS can resist at different magnetic field strengths (from 30 to 100 mT). The solid line is USMS with MN, and the dashed line is USMS without MN. (c) The USMS achieves targeted anchoring to the tissue model under flowing fluid at a velocity of 201 mm/s. (d) Schematic images of USMS remaining anchored under intestinal peristalsis, where the USMS is subjected to forces from the intestine (*F*_1_ and *F*_2_) and anchoring forces (*F*_a_). Robust anchoring of the USMS on the target tissue under intestinal peristalsis is monitored by endoscopy. (e) The USMS stably anchors in the rat digestive tract. As the microneedle separates from the magnetic substrate, it can be excreted through the digestive tract. (f) Wrap-around penetration and on-demand drug delivery in rat stomach. Fluorescence microscopy images of tissue frozen sections demonstrate microneedle penetration and drug delivery in deep tissue. Tissue sections of the rat stomach and intestine demonstrate no leakage of magnetic particles and tissue damage during excretion. Scale bars: (a–e) 5 mm, (f) 100 μm.

The digestive tract is typically a complex dynamic environment that includes liquid flow and peristalsis. Therefore, robust anchoring of microneedle devices in this dynamic environment is critical for practical applications. Benefiting from bending magnetization, USMS not only ensures its wrap-around penetration of 3D tissues but also improves its ability to anchor on-demand regions in complex environments (Fig. S31). As shown in Fig. 5a, the ability of the USMS to counteract shear force is tested. The higher the magnetic field strength, the more shear force the USMS can counteract, increasing from 23.4 mN at 30 mT to 66.5 mN at 100 mT (Fig. 5b and Movie S5). In addition, programmable magnetized USMS also demonstrates strong anchoring capability under a dynamic environment. USMS demonstrates strong anchoring capability under different liquid flows, ranging from 134 to 402 mm/s (Fig. S32 and Movie S6). Due to strong magnetic response characteristics, the USMS can move against fluid flow (201 mm/s) and achieve anchoring of the tumor model (Fig. 5c). The peristalsis force (F_1_ and F_2_) generated from the intestine is composed of compressive force (20 mmHg) and shear force (0.1–1 N/cm^2^) [59,60]. To verify the feasibility of the USMS application for the treatment of actual intestinal tissues, its anchoring stability under intestinal peristalsis is tested (Fig. S33). As shown in Fig. 5d, the USMS anchoring target under intestinal peristalsis is demonstrated by real-time endoscope imaging (Movie S6). USMS remains anchored in the target tissue under 3 cycles of intestinal peristalsis [61]. Finally, we validate the feasibility of robust anchoring and on-demand drug delivery of USMS in the rat digestive tract. As shown in Fig. S34, a magnet with a radius of 15 mm is wrapped around the right side of the rat’s abdomen with the medical bandage. The magnet is ∼10 mm away from the USMS in the rat’s stomach, where the measured magnetic flux density is 57.4 mT for reliable USMS operation in vivo (Fig. S35). USMS stably anchors in the rat’s digestive tract without displacement over 3 days (Fig. 5e). As the magnetic substrate separates from the microneedle, it can be excreted with the rat’s feces, which avoids the metabolic toxicity of NdFeB residues to the rat. At the same time, on-demand drug delivery has been validated. As shown in the cryosection results, significant penetration is achieved in the rat digestive tract (Fig. 5f and Fig. S36). Red fluorescence also confirms the on-demand release of DOX drug molecules in rat tissues. Moreover, USMS did not produce any leakage of magnetic particles during excretion from the digestive tract and did not cause any damage to the intestinal tract and other rat tissues (Fig. S37). In short, the USMS with wrap-around penetration demonstrates the robust anchoring capability to face liquid flow and intestinal peristalsis and finally achieves on-demand drug release in target regions.

### USMS monitors tumor lesion size through ultrasound imaging

**Figure 6.**
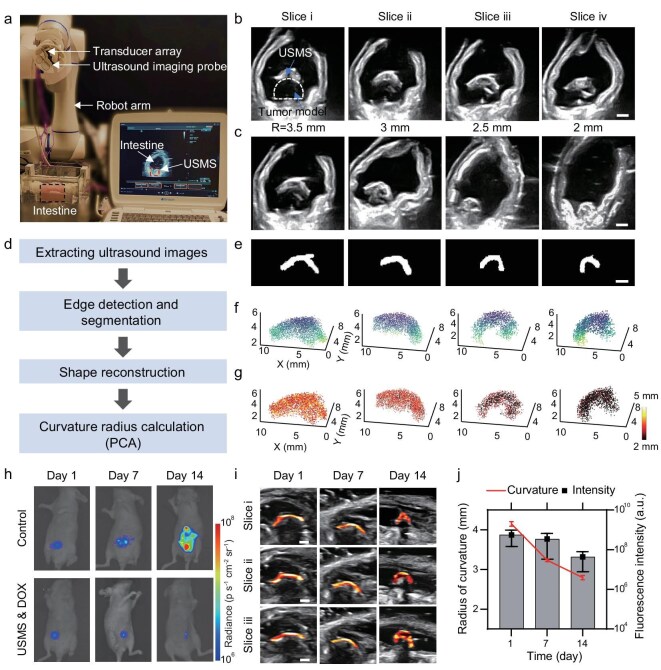
USMS continuously monitors tumor lesion size through sensitive ultrasound imaging. (a) The setup for sensing lesion size in an *in vitro* intestinal model based on the shape morphing and anchoring of USMS. (b) Ultrasound images of USMS-wrapped tumor models at different scanning sequences (i–iv). Compared to the tissue model, USMS exhibits strong ultrasound reflection. (c) Ultrasound images of USMS-wrapped tumor models with different radii (2–3.5 mm). (d) Schematic diagram of the process of inferring the size of the wrapped tumor model from ultrasound images, including extracting ultrasound images, edge detection and segmentation, shape reconstruction, and curvature radius calculation. (e) Results of USMS ultrasound image segmentation and extraction. (f) 3D USMS point cloud map reconstructed from the segmented image. (g) The curvature radius of USMS is calculated from the reconstructed 3D point cloud map. (h) Representative *in vivo* fluorescence images of mice under two groups (control and USMS&DOX) at different points post-treatment. (i) Ultrasound images of USMS at various time points during USMS&DOX treatment, illustrating real-time tumor size monitoring capability. (j) Quantitative comparison of fluorescence intensity and ultrasound sensing radius, where fluorescence and radius decrease over time. Error bars represent the standard deviation (*n* = 3). Scale bars: 2 mm.

In addition to targeted drug delivery, robust anchoring and long-term sensing of therapeutic outcomes are crucial for effective disease treatment. However, achieving long-term, non-invasive sensing of the therapeutic effect *in situ* remains challenging. Colon cancer, as a common malignant tumor of the digestive tract, is selected to evaluate USMS’s functions. Benefiting from its excellent anchoring capability and strong ultrasound imaging performance, the USMS enables real-time feedback of tumor size changes after treatment, thereby facilitating therapeutic evaluation. To this end, we developed a tumor assessment system (Fig. 6a) composed of a robotic arm, an image acquisition card, and an ultrasound imaging device. The robotic arm manipulates the ultrasound probe to perform customized scanning and acquire multi-angle images of the USMS, enabling reconstruction of its 3D structural features. As illustrated in Fig. 6b, sequential ultrasound images from different cross-sections are collected. Due to its higher ultrasound reflectivity compared to the tumor model, the USMS serves as a visible marker, allowing accurate evaluation of the encapsulated tumor size. To validate this method, we prepared hemispherical tumor models with radii of 2.0, 2.5, 3.0, and 3.5 mm (Fig. 6c), each encapsulated by a USMS. The assessment workflow (Fig. 6d) includes ultrasound image extraction, edge detection and segmentation, 3D shape reconstruction, and curvature radius calculation. Figure 6e presents processed ultrasound images of the USMS after segmentation. Sequentially acquired image slices are used to reconstruct the 3D point cloud of the USMS covering tumors of various sizes (Fig. 6f). Furthermore, using principal component analysis, the local curvature radius of the USMS surface is calculated to estimate the size of the underlying tumor model (Fig. 6g). USMS covered lesions of different sizes and presented different curvature radii under ultrasound imaging, which were 2.13, 2.52, 3.03, and 3.42 mm, respectively. The results show that the curvature radius obtained by ultrasound image processing was highly consistent with the actual tumor radius, with an average error of 2.6%. Therefore, the developed USMS provides a non-invasive, real-time, and repeatable strategy for long-term assessment of tumor treatment efficacy.

To validate the therapeutic efficacy, anchoring capability, and long-term sensing potential of USMS *in vivo*, an orthotopic colorectal tumor model was established in nude mice. As shown in Fig. S38, HCT 116 colon cancer cells were orthotopically injected into the lateral wall of the mouse cecum. After a 10-day incubation period, well-established tumors were formed. Drug-loaded USMSs were delivered to the tumor site, and targeted drug release was triggered by focused ultrasound irradiation. To assess therapeutic efficacy, bioluminescence imaging and ultrasound imaging were performed on days 1, 7, and 14. The implanted HCT 116 cells were stably transfected with luciferase (Luc), enabling quantitative fluorescence sensing of tumor progression. As shown in Fig. 6h, fluorescence signals of control and treatment groups were recorded at multiple time points. Quantitative analysis revealed that the USMS&DOX group exhibited a significant reduction in total tumor fluorescence intensity over time, with the strongest therapeutic response observed on day 14, compared to the control and other treatment groups. In parallel, ultrasound imaging was used to track USMS morphology *in vivo* (Fig. 6i). USMS showed distinct ultrasound contrast, allowing clear visualization throughout the treatment period. Notably, the curvature radius of the USMS gradually decreased over time, from 4.2 mm on day 1 to 2.8 mm on day 14, which correlated well with the decrease in tumor size inferred from fluorescence imaging (Fig. 6j). After 14 days, the USMS magnetic substrate showed no weight change and NdFeB leakage, demonstrating its temporal stability and biocompatibility *in vivo* (Fig. S39). At the end of the treatment cycle, the colorectal tumors were harvested and subjected to histopathological analysis. As shown in Figs S38 and S40, hematoxylin–eosin staining and immunofluorescence revealed a significant reduction in proliferative marker Ki67 and a marked increase in apoptotic marker TUNEL in the USMS&DOX group. Statistical analysis further confirmed that Ki67 expression was markedly reduced, while TUNEL positivity was highest in the USMS-treated group, indicating pronounced suppression of tumor cell proliferation and induction of apoptosis. Histological and immunofluorescence analyses confirmed that systemic DOX administration did not produce significant tumor suppression compared with the control group (Fig. S41). These results demonstrate that the USMS platform achieves precise drug delivery and efficient tumor ablation, while simultaneously enabling morphological monitoring of tumor dynamics via curvature radius analysis. This integrated therapeutic and diagnostic capability makes USMS a promising candidate for minimally invasive treatment and long-term sensing of intraluminal malignancies.

## DISCUSSION

Compared with traditional medical endoscopes, our USMS has advantages in many aspects, such as preoperative preparation, invasiveness, reachable area, cost, and long-term monitoring capabilities (Table S4). In summary, to address the challenges of remote and long-term lesion assessment in complex digestive tracts, we developed a bioinspired USMS with high ultrasound sensitivity, controllable locomotion, robust anchoring, and on-demand drug release. The USMS has shown substantial advantages in the following aspects. First, the USMS presents strong ultrasound sensitivity for navigation and long-term tumor size sensing *in vivo*, facilitating lesion size assessment. Second, the USMS shows controllable locomotion and wrap-around penetration, leading to the USMS exhibiting robust anchoring capability to overcome liquid flow and peristalsis in the digestive tract. Last, the USMS’s treatment and efficacy assessment was rigorously validated through comprehensive *in vivo* experiments using animal models, which lays the foundation for future practical applications. The USMS presented here will promote the potential applications of microneedles and microrobots for targeted treatment in the digestive tract. To distinguish our USMS from other devices, we compared various representative miniature medical devices for drug delivery in the digestive system, including active targeting, shape morphing, anchoring capabilities, active drug release, and therapeutic efficacy sensing (Table S5).

USMSs demonstrate advantages in on-demand delivery, robust anchoring, and long-term lesion assessment in the digestive tract, which will attract extensive research. For the digestive tract environment full of wrinkles and bubbles, oral defoaming solution and optimization of the USMS shape morphing capability using origami design can further improve its ultrasound sensing accuracy and robustness. In addition, USMSs could also load the other cargos, such as vaccines [62], DNA [63], or insulin [64], etc., leading to being used for immunotherapy and decreasing blood sugar. Currently, the substrate of USMS consists of an undegradable elastomer and NdFeB MPs, which should be replaced by degradable hydrogels and other biocompatible magnetic nanoparticles in the future [65,66]. In future iterations, the USMS substrate could be replaced with degradable hydrogel matrices (e.g. PEGDA or alginate-based systems) integrated with biocompatible magnetic nanoparticles such as Fe₃O₄, enabling tunable degradation kinetics and improved biosafety while maintaining magnetic responsiveness. Furthermore, the present research is limited to isolated tissue and small animals. To assess the application feasibility of our proposed USMS, it is preferable to select large animals such as pigs and sheep in the future. Thick tissue in large animals makes it difficult for magnetic fields and ultrasound to achieve functionalities. Fortunately, the depth of focus of the ultrasound field can be adjusted by the number and spatial arrangement of ultrasound transducers. In addition, commercial magnetic field generators are sufficient to generate a strong magnetic field strength (>50 mT) for USMS motion and anchoring in large animal and human digestive tracts. As a next step, we will discuss with clinicians to understand the clinical problems and sensing requirements of diseases to optimize the USMS. This work promotes potential collaboration among materials science, engineering, and clinical medicine, thereby facilitating the clinical translation of USMSs.

## MATERIALS AND METHODS

### Materials

The chemicals of triethoxy (1H,1H,2H,2H-perfluoro-1-octyl) silane (POTS), dichloromethane (>99%), PLGA (Mn = 2000), PVP (Mn = 1 300 000), dimethyl sulfoxide (DMSO, >99%), and gelatin were purchased from Advanced Technology and Industrial Co., Ltd. The PEGDA is obtained from (BMF Material Technology Inc., Shenzhen, China). Calcein AM, propidium iodide (PI), DAPI, and Hoechst 33 258 were purchased from Beijing Soleibao Technology Co., Ltd (Beijing, China). The DPHA was purchased from Curease Chemical Co., Ltd (Shanghai, China). Ecoflex 00–30 was purchased from Smooth-on Inc. The other chemicals were purchased from Aladin (China). All reagents were used without any treatment. Deionized water with a resistivity of 18.2 MΩ cm subjected to ultraviolet sterilization was used for the preparation of all aqueous solutions.

### Fabrication of USMS

MNs made of PEGDA resin were cured under 405 nm ultraviolet light and printed with a precision of 2 μm by a high-resolution 3D printer based on surface projection micro stereolithography. The 3D model of the MN was first built with SolidWorks 2021 software, then imported into the BMF 3D printer driver software for printing. The magnetic elastomers were prepared by blending silicone-based materials (Ecoflex 00–30 parts A and B) and NdFeB magnetic microparticles (5 μm) in a 1:1 mass ratio. After degassing, the magneto-elastomer was cured in an oven (50°C) for 30 min. Finally, the substrate was taken out from the mold by the demolding process. The bonding material PLGA was first dissolved in dichloromethane (10 wt%), then coated on the surface of the magnetic substrate with a spatula, and then MN was pressed on the surface, and a tight connection was formed after waiting for 5 min at room temperature.

### Characterization techniques

The morphology of MNs is characterized by SEM (JEOL 7800F). The magnetic properties are evaluated at 300 K using a PPMS Model 6000 Quantum Design VSM. The release of DOX is measured by UV–Vis spectrophotometer (Hitachi U2910). The fluorescence spectra and cell viability are recorded by a microplate reader (Tecan Infinite M Plex). The fluorescence images are captured with an inverted fluorescence microscope (Nikon Eclipse Ti). The mechanical properties of the magneto-elastomers, microneedles, and USMSs were measured by a mechanical tester (MACH-1 mechanical tester v500cst, MA008). Tissue fluorescence images captured by Perkin Elmer In Vivo Imaging System.

Detailed methods are provided in the online supplementary material.

## Supplementary Material

nwag179_Supplemental_Files
