# Supplementary material for: Ultrasound-sensitive microrobotic sensor with robust anchoring for long-term digestive lesion assessment
Source: Natl Sci Rev. 2026 Mar 20;13(10):nwag179. doi: 10.1093/nsr/nwag179 (PMC13196706; doi:10.1093/nsr/nwag179)
Supplement: nwag179_Supplemental_Files [file nwag179_supplemental_files.zip › Supplementary data.pdf]

Supplementary Materials for

Ultrasound-sensitive microrobotic sensor with robust anchoring for long-term digestive lesion assessment

Chen Xin[1,2,†], Yuqiong Wang[2,†], Yihang Jiang[2,†], Bo Hao[2], Neng Xia[2], Jiaqi Zhu[2], Lin Su[2,*], Jinsheng Zhao[2], Xin Wang[2], Haojin Yang[2], Xianfeng Xia[3], Kai Fung Chan[4,*], Qingsong Xu[5,*], Dong Wu[1,*], Philip Wai Yan Chiu[3,4], Joseph Jao Yiu Sung[6] and Li Zhang[2,3,4,*]

[1]Department of Precision Machinery and Precision Instrumentation, University of Science and Technology of China, Hefei 230026, China;
[2]Department of Mechanical and Automation Engineering, The Chinese University of Hong Kong, Hong Kong 999077, China;
[3]Department of Surgery, The Chinese University of Hong Kong, Hong Kong 999077, China;
[4]Chow Yuk Ho Technology Center for Innovative Medicine, The Chinese University of Hong Kong, Hong Kong 999077, China;
[5]Department of Electromechanical Engineering, Faculty of Science and Technology, University of Macau, Macau 999078, China;
[6]Lee Kong Chian School of Medicine, Nanyang Technological University, Singapore 308232, Singapore
[*]**Corresponding authors**. E-mails: sulin@link.cuhk.edu.hk; kaifungchan@cuhk.edu.hk; qsxu@um.edu.mo; dongwu@ustc.edu.cn; lizhang@cuhk.edu.hk

[†]Equally contributed to this work.

Methods

Magnetic force calculation

Theoretically, by using the Euler-Bernoulli equation, the torque balance equation for an arbitrary infinitesimal element can be expressed as [1],

$$\boldsymbol{\tau}_m + \int_s^L \frac{F_y}{A} ds \cos\theta - \int_s^L \frac{F_x}{A} ds \sin\theta = -\frac{EI}{A}\frac{\vartheta^2\theta}{\vartheta s^2} \qquad \text{S(1)}$$

$$\boldsymbol{\tau}_m = \begin{bmatrix} 0 & 0 & 1 \end{bmatrix} \left\{ \begin{bmatrix} \cos\theta & -\sin\theta & o \\ \sin\theta & \cos\theta & o \\ 0 & 0 & 1 \end{bmatrix} \times \boldsymbol{B} \right\} \qquad \text{S(2)}$$

F_x and F_y denote the magnetic force applied along the *x*-axis and *y*-axis, respectively (Supplementary note S2). The variables τ_m and A represent the magnetic torque (per volume) and cross-sectional area. The variables *E*, *I*, *s*, and *L* denote Young's modulus, cross-sectional moment of area, the length of is the length of the deformed magnetic arm, and the total length of the magnetic arm, respectively. Based on the above equation, the deformation degree and shape of the USMS under magnetic force can be predicted.

Integration of the magnetic actuation system with the medical imaging system

The system consists of a magnetic drive system and an ultrasound imaging system. A permanent magnet (25 mm in diameter) is mounted on a motor using a 3D printed fixture, and the motor is gripped by a robot arm. The robot arm and motor were controlled by a LabVIEW program via myRIO (National Instruments). An ultrasound system (Terason t3200, Teratech Inc., USA) was integrated into the magnetic drive system to image the USMS. A linear array probe (16HL7, Teratech Corporation, USA) with a bandwidth of 7 to 16 MHz was mounted on a 3-DoF robotic manipulator. It was driven by an Arduino Mega and controlled using a LabVIEW program. During navigation, ultrasound images are acquired in real time. Like the integrated systems described above, endoscope (COLA BVET-0603, TOW-INT TECH, Shanghai) and X-ray (c-arm fluoroscopy, Aritis Zeego, Siemens) imaging systems were used to monitor the operation process instead of optical microscope.

Tissue model construction and drug therapy

The Murine Carcinoma-38 (MC-38) cells were cultured in normal Dulbecco's modified eagle medium (Gibco, Thermo Fisher Scientific, Grand Island, NY) supplemented with 10% fetal bovine serum (HyClone, Logan, UT) and 1% penicillin/streptomycin (Gibco, Life Technologies, Grand Island, NY). For tissue model construction, MC-38 cells were detached from the culture dish by trypsinization with 0.25% Trypsin-EDTA (Gibco, Thermo Fisher Scientific, Grand Island, NY) at 37°C for 30 s and centrifuged at 1000 rpm for 5 min. Then, the cells were resuspended in culture medium at a density of 2×10^6 mL^{-1} for use. The hydrogel section is composed of 10 wt% GelMA, 0.5 wt% lithium phenyl-2,4,6-trimethylbenzoylphosphinate in DI water. Subsequently, MC-38 cells were mixed with hydrogel precursors to construct different shapes of tissue models using extrusion bio-3D printers (Biomaker1, SUNP, Beijing) or inverted mold technology. The following four experimental groups were included across all relevant in vitro and ex vivo drug delivery experiments. (I) Control group: without USMS

and DOX delivery. (II) USMS group: USMS without DOX loading, used to evaluate the effect of mechanical & acoustic stimulation alone. (III) Local DOX group: local DOX delivery without USMS-mediated activation, serving as a baseline for passive drug diffusion. (IV) USMS&DOX group: DOX-loaded USMS activated by ultrasound, enabling ultrasound-triggered and sustained drug release. After each treatment, the hydrogels were stained using a live/dead fluorescence assay to assess cell viability. Specifically, 4',6-diamidino-2-phenylindole (DAPI) was used to label the nuclei of all cells, emitting blue fluorescence and indicating total cell count. Calcein Acetoxymethyl Ester (AM) was used to label viable cells, which emitted green fluorescence due to intracellular esterase activity, while Propidium Iodide (PI) stained the nuclei of dead or membrane-compromised cells, appearing red under fluorescence imaging.

Gelatin (200 mg) was first dissolved in DI water (1 mL) at 40°C. Agar (5 mg) was dissolved in DI water (1 mL) at 100°C. Subsequently, the above two solutions are mixed through stirring 3 h. Adriamycin hydrochloride (DOX) was added to the mixed hydrogel precursor. The drug-filled gelatin was finally instilled into a microneedle hollow channel for drug release.

Ultrasound triggering of USMS

The lead zirconate titanate (PZT) piezoelectric transducers, each measuring 20 mm in diameter and 1.2 mm in thickness, are arranged on a spherical surface with a diameter of 90 mm. This configuration enables the acoustic waves generated by the transducers to converge and focus on the center point of the sphere, functioning as the focal point of the acoustic field. The transducers operate in their first-order longitudinal vibration mode at a resonance frequency of 1.7 MHz.

***In vivo* animal experiments**

Male rats used in the experiment were food-restricted for 24 hours before the procedures. We implant the USMS in the rat's stomach and then suture the wound. Permanent magnets are tied to the abdomen of rats with medical bandages for in vivo anchoring of USMS.

To establish an orthotopic tumor model, 7-week-old female BALB/c-nude mice were anesthetized. A small incision was made to expose the cecum. HCT 116 cells (6×10^6) suspended in 30 μL of PBS were carefully injected into the serosa of the cecum. Following the injection, the incision was sutured. The ethical approval from the Institutional Animal Care and Use Committee was obtained before animal research (TOP-IACUC-2024-0328).

Note S1. The shape and size design of the ultrasound-sensitive microrobotic sensor (USMS)

In our work, the USMS's shape is derived from a hemisphere. As shown in Fig. S1, a hemisphere could be divided into several fan-shaped patterns. The sum of the fan-shaped pattern width is equal to the circumference of the cross-sectional circle. In addition, its length is equal to half of the circumference. Here, we set the semicircle radius to 2 mm. To ensure the bending of the USMS, the length (*l*) of each patch is smaller than 2.5 mm. When the hemisphere is divided into N planes, the width of each plane is calculated as

$$w = \frac{2\pi r}{N} \quad \text{S(3)}$$

Since the distance between the microneedle to the edge is 0.4 mm and the distance between two microneedles is 0.7 mm, the number of microneedles could be calculated.

$$N_M = N * \frac{(w-1.1)}{0.7} * \frac{(l-1.1)}{0.7} \quad \text{S(4)}$$

Thus, the number of microneedles in different designs is compared in the table below.

Table S1 The microneedle quantities of different designs.

N	w	l	N_M
3	4.19	2.5	45
4	3.14	2.5	36
5	2.51	2.5	45
6	2.09	2.09	24

In summary, to ensure the maximum number of microneedles, we chose a USMS design with five microneedle patches.

Note S2. Magnetic force calculation of USMS.

USMS is applied in a 3D mold for magnetization, where the radius of the magnetization circle is R_0=2 mm. Therefore, we can calculate the magnetization intensity at point P under a uniform magnetic field B=2 T by formula S1.

$$M = \begin{cases} M_t = M_0 \sin\theta \\ M_n = M_0 \cos\theta \end{cases} \quad \text{S(5)}$$

The saturation magnetization (M_0) of each patch is 214087 A/m. When the magnetic substrate is placed flat, its magnetization strength is expressed by,

$$\begin{cases} M_x = M_t = M_0 \sin\theta \\ M_y = M_n = M_0 \cos\theta \end{cases} \quad \text{S(6)}$$

Subsequently, when an external magnetic field is applied, the magnetic substrate is subjected to forces as shown in Fig. S15. The component of the magnetization intensity at the deformed point P extending in the direction of the x-coordinate axis is,

$$M_x' = M_t cos\alpha - M_n sin\alpha = M_0 \sin\left[\left(1 - \frac{r_0}{r_1}\right)\theta\right] \quad \text{S(7)}$$

Since the arc length from point P to the base center Q remains constant,

$$r_0 \times \theta = r_1 \times \alpha \qquad \text{S(8)}$$

Thus, when USMS is actuated by a magnetic field (B_0), the combined moment τ can be calculated as,

$$\tau = \int_0^{\theta_0} M_x' \cdot B_0 \cdot S_1 \cdot r_0 \, d\theta + \int_{\theta_0}^{\theta_1} M_x' \cdot B_0 \cdot S_2 \cdot r_0 \, d\theta$$

$$= \frac{M_0 B_0 r_0 s_1}{1 - r_0/r_1}\{1 - cos[(1 - r_0/r_1)\theta_0]\} + \frac{M_0 B_0 r_0 s_2}{1 - r_0/r_1}\{cos[(1 - r_0/r_1)\theta_0] - cos[(1 - r_0/r_1)\theta_1]\} \qquad \text{S(9)}$$

Where S_1=0.5 mm^2 and S_2=1.25 mm^2 are the cross-sectional areas of different locations of the substrate, respectively.

Finally, the moment of the magnetic field applied to the substrate is equal to the resistance moment applied to it by the object,

$$F \cdot cos\, {}^{\gamma}\!/_{2} \cdot L_2 = \tau \qquad \text{S(10)}$$

$$F = \frac{\tau}{cos^{\gamma}/_{2} \cdot 2 r_1 \cdot sin^{\gamma}/_{2}} \qquad \text{S(11)}$$

Where $\gamma = r_1/L_1$ is the angle between the O point and the Q point. Arc length 1 (L_1) and Arc length 1 (L_2) are 1.72 mm and 4.5 mm. Based on the above-mentioned parameters, we can calculate the magnetic moments generated by the magnetic substrate at different magnetic field strengths (22.5 mT, 30 mT, 36 mT, 50 mT, and 75 mT). Therefore, the magnetic force can be calculated from equation S (6). All parameter values are used for magnetic force calculation (Table S2). A comparison of theoretical calculations and experimental testing of the magnetic force is shown in Table S3.

Table S2 Parameter values used in the theoretical calculation of magnetic force.

Parameters	Value	Unit
Saturation magnetization(M_0)	214087	A/m
Area of region 1 (S_1)	0.5	mm^2
Area of region 2 (S_2)	1.25	mm^2
Magnetization circle radius (R_0)	2	mm
Target radius (R_t)	3.5	mm
Arc length 1 (L_1)	1.72	mm
Arc length 2 (L_2)	4.5	mm

Table S3 Comparison of theoretical calculations and experimental testing of magnetic force.

Magnetic strength (mT)	Magnetic force (calculation, mN)	Magnetic force (measurement, mN)
22.5	3.882	4.681
30	5.176	5.796
36	6.211	7.282
50	8.627	9.066
75	12.941	11.295

Note S3. Mechanical performance testing of USMS and artificial hydrogel.

For hydrogels and microneedles, we are testing Young's modulus using a cylinder with a radius (r) of 0.35 mm in a compressive manner. Young's modulus can be expressed as,

$$E = \frac{T(1-\mu^2)}{2r} \quad S(12)$$

Where T is the slope of the plot, μ (0.35) is Poisson's ratio.

For magnetic polymer substrates, Young's modulus is tested by stretching. The original length, width, and height of the magnetic polymer are 26 mm, 5 mm, and 0.226 mm. The polymer is stretched by 1 mm during the test. Thus, the Young's modulus can be calculated as,

$$E = \frac{F/S}{\Delta L/L} \quad S(13)$$

Finally, Young's modulus of hydrogel and magnetic polymers are 11.16 kPa and 128 kPa.

Table S4 Comparison of endoscope and our miniature robot for GI tract delivery and imaging.

Device	Preoperative anesthesia	Professional hospital & doctor	Invasiveness	Long-term monitoring	Cost
Endoscope	Yes	Yes	Relatively high	No<br>(~1 h)	High<br>(1000~5000 USD)
USMS	No	No	No	Yes<br>(ultrasound, 2 weeks)	Low<br>(<10 USD)

Table S5 Comparison of various miniature medical devices for drug delivery in the digestive tract. To distinguish our USMS from others, we compared various representative miniature medical devices for drug delivery in the digestive tract, focusing on motion, shape morphing, robust anchoring, on-demand drug delivery, and long-term monitoring. N.A. represents not applicable.

**Motion**		**Shape morphing**		**Robust anchoring**	**On-demand drug delivery**		**Long-term monitoring**		Ref.
Strategy	Actuation source	Actuation source	Morphing numbers	Anchoring	Drug release modes	Drug release positions	In situ	Time	
Passive	No	Liquid	One	N.A.	Degradation	One non-specific position (intestine)	No	N.A.	[2]
	No	Temperature	One	9~24 h	Dissolution	One non-specific position (colon)	No	N.A.	[3]
	No	N.A.	N.A.	N.A.	Dissolution	One non-specific specific position (stomach)	No	N.A.	[4]
	No	Spring	One	N.A.	Dissolution	One non-specific specific position (intestine)	No	N.A.	[5]
Active	Single (Magnetic)	Magnetic	Reversible	N.A.	N.A.	N.A.	No	N.A.	[6]
	Single (Magnetic)	pH	One	N.A.	Dissolution	N.A.	No	N.A.	[7]
	No	N.A.	N.A.	N.A.	pH triggering	Non-specific positions (intestine)	No	N.A.	[8]
	Single (Magnetic)	N.A.	N.A.	N.A.	Degradation	One specific position (intestine)	No	N.A.	[9]
	**Dual (Magnetic and ultrasound)**	**Magnetic**	**Reversible**	**14 days, 402 mm/s liquid**	**Focused ultrasound**	**Multi-Specific positions (3 positions)**	**Yes**	**14 days**	This work

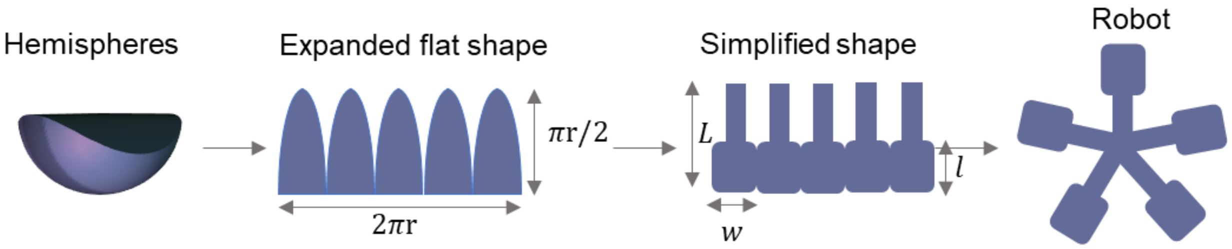

Fig. S1 The dimensional design of the USMS is derived from the expanded flat shape of the hemisphere structure.

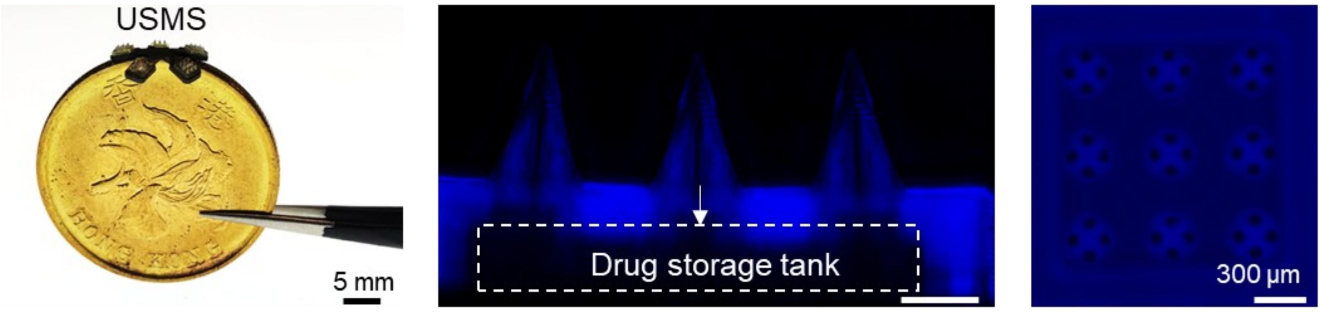

Fig. S2 Images of the magnetic USMS (size <10 mm), fluorescence images showing hollow channels and drug storage tanks.

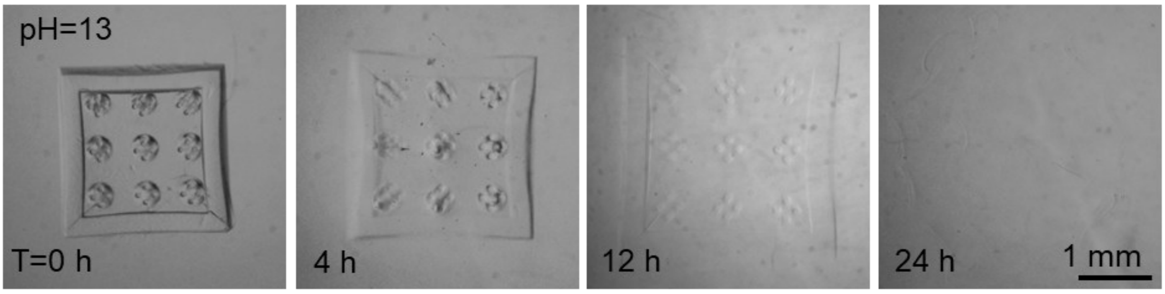

Fig. S3 The degradation process of microneedles during liquids with pH =13.

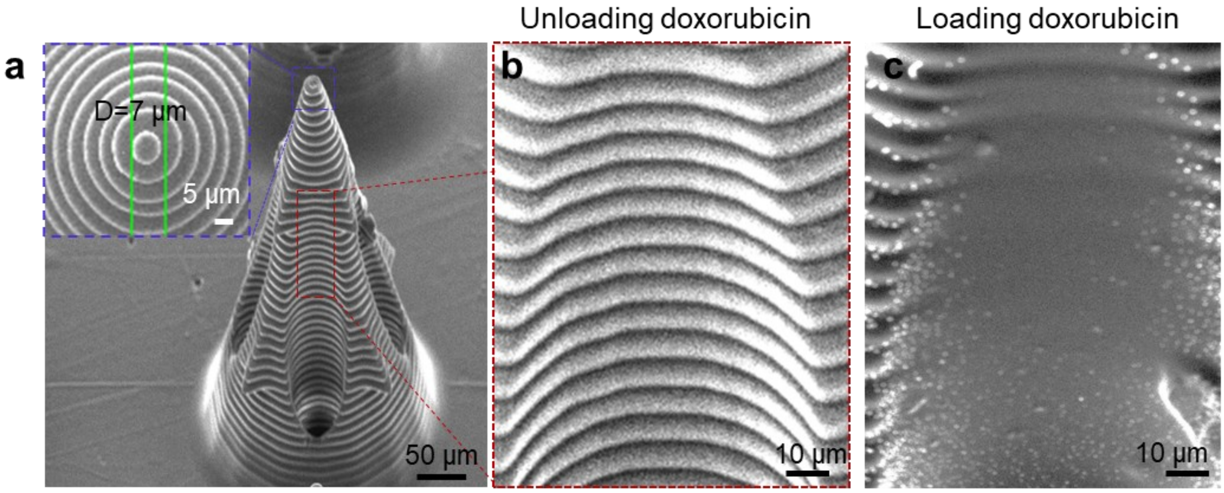

Fig. S4 SEM images of a microneedle (MN). (**a**) The tip of the MN is ~7 µm. (**b**) Enlarged image of the MN showing layer-by-layer stacking traces. (**c**) Enlarged image showing hydrogel-filled doxorubicin (DOX) loaded in the hollow channel.

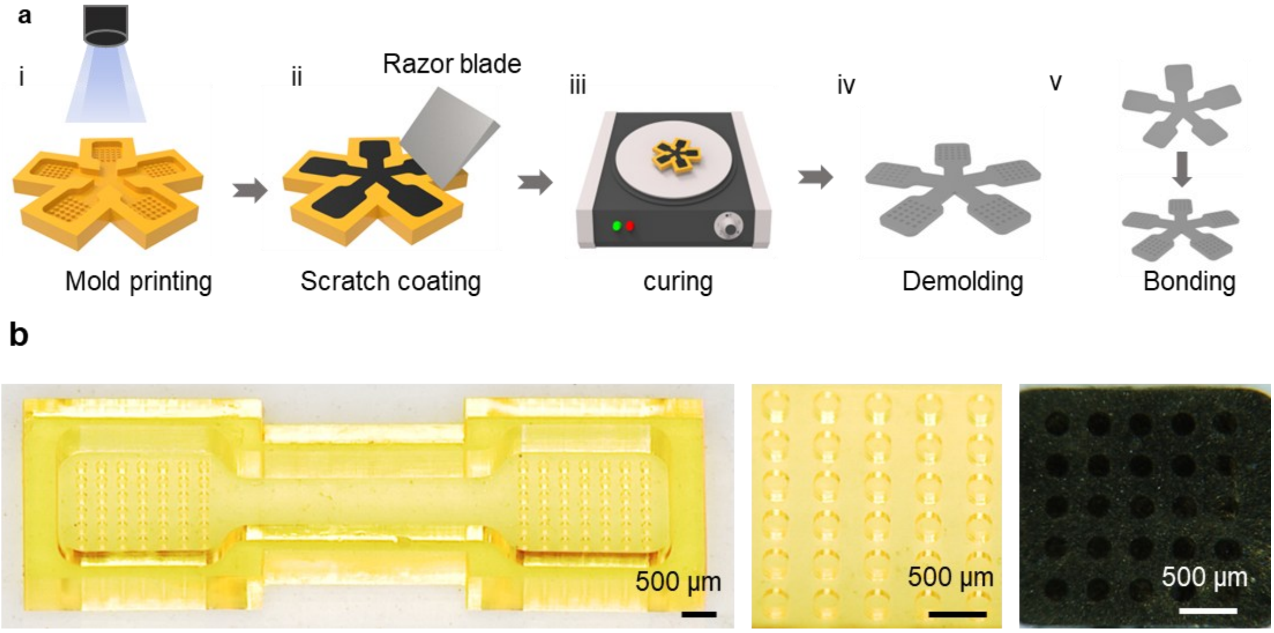

Fig. S5 **(a)** Schematics of the fabrication process of the magnetic substrate. (i) Schematic of 3D printing the mold using the micro stereolithography (µSLA). (ii) Illustration of fabricating a ferromagnetic-elastic substrate with a desired thickness. The mixture of Ecoflex-0030 elastomer and NdFeB microparticles is poured onto a polymer substrate. A razor blade is used to scratch against the spacer for a uniform thickness. (iii) The samples are baked on a hot plate (70°C) for 1 hour for curing. (iv) Illustration of the magnetic substrate after demolding. **(b)** Images of molds and substrates with multiple air cavities to enhance ultrasound sensitivity for long-term lesion assessment *in vivo*.

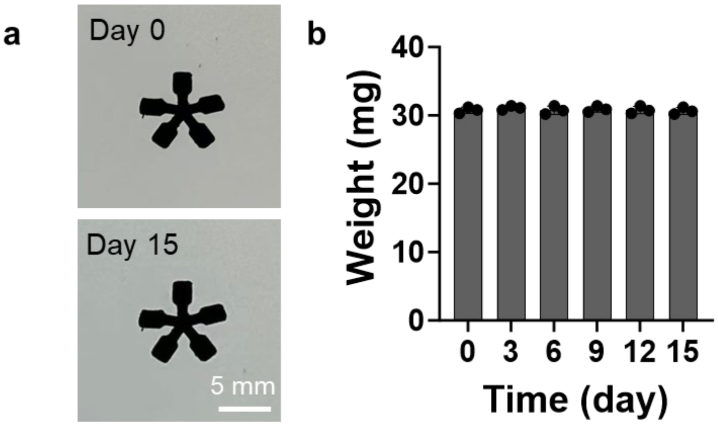

Fig. S6 Structural integrity assessment of the magnetic substrate after prolonged exposure. (**a**) Representative optical images of the magnetic structure at Day 0 and Day 15 following immersion or in vivo exposure. No visible structural deformation or fragmentation was observed. (**b**) Quantitative mass measurements showed no significant weight change over the 15-day period, indicating preserved structural stability. Error bars represent the standard deviation (n = 3).

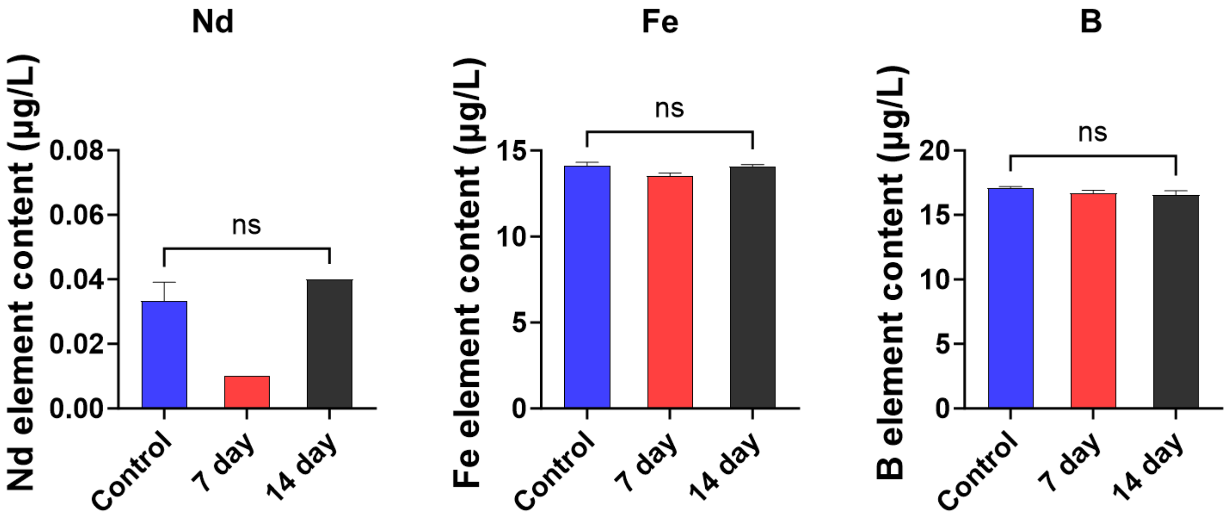

Fig. S7 Elemental analysis of Nd, Fe, and B under different conditions. Concentrations of neodymium (Nd), iron (Fe), and boron (B) were measured in control (artificial intestinal fluid), 7-day (artificial intestinal fluid after 7-day immersion of the magnetic substrate), and 14-day groups (artificial intestinal fluid after 14-day immersion of the magnetic substrate). No significant differences were observed among groups. Error bars represent the standard deviation (n = 3).

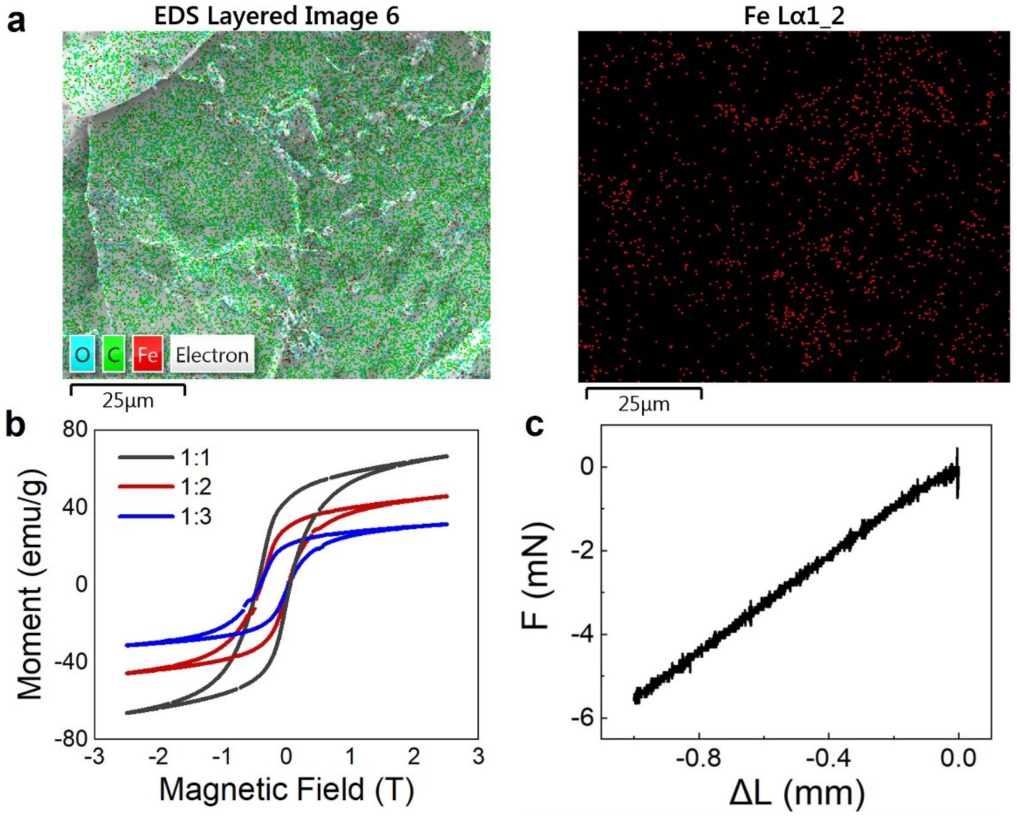

Fig. S8 Magnetic substrate consists of NdFeB microparticles (MPs) and soft polymer. (**a**) EDS images of the substrate, which is mainly composed of C, O, and Fe. (**b**) Hysteresis curves of polymers with different MPs content. (**c**) The mechanical properties of the magnetic polymer are tested, and Young's modulus is calculated as 128 kPa.

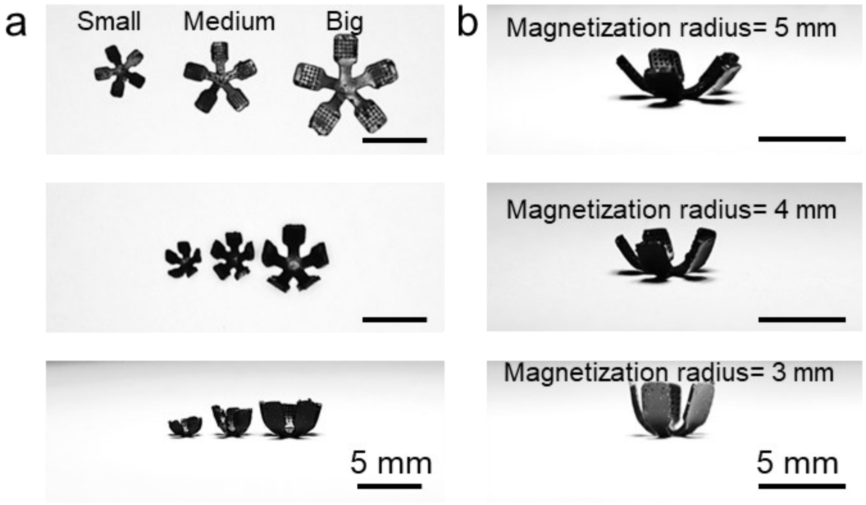

Fig. S9 Customization of soft substrate size (**a**) and magnetization radius (**b**). In this way, the USMS could match targets of different sizes.

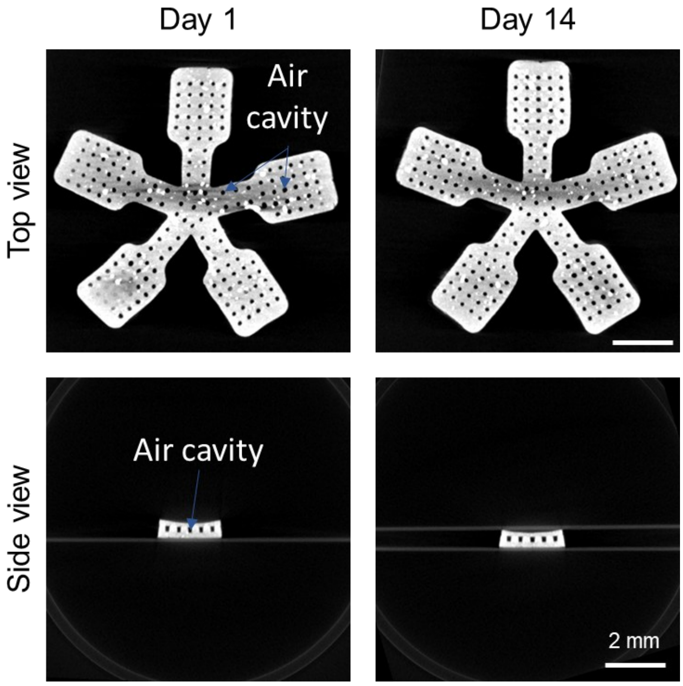

Fig. S10 Micro-CT characterization of the integrated air-cavity structure. Representative micro-CT images showing top-view (upper panels) and cross-sectional (lower panels) reconstructions of the microrobotic structure containing internal air cavities. The radiolucent regions correspond to the enclosed air cavities. No structural collapse or deformation of the cavity architecture was observed.

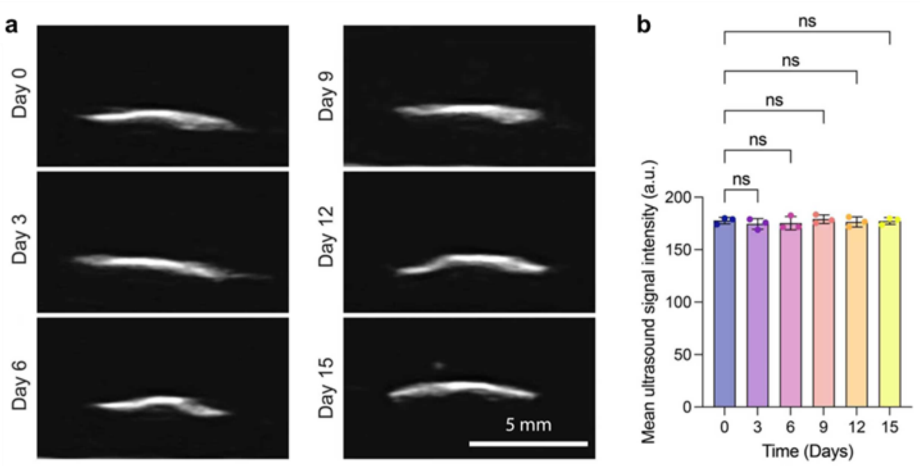

Fig. S11 Long-term stability of ultrasound reflectivity from air-cavity structures. (**a**) Representative ultrasound images of the air-cavity structure acquired at Days 0, 3, 6, 9, 12, and 15 under physiologically relevant conditions (37 °C, artificial intestinal fluid). The reflected signal morphology and intensity remain visually consistent throughout the 15-day period. (**b**) Quantitative analysis of mean ultrasound signal intensity over time. Signal intensity was normalized to Day 0. No statistically significant differences were observed between time points, indicating negligible attenuation of acoustic reflectivity during the testing period. Error bars represent the standard deviation (n = 3).

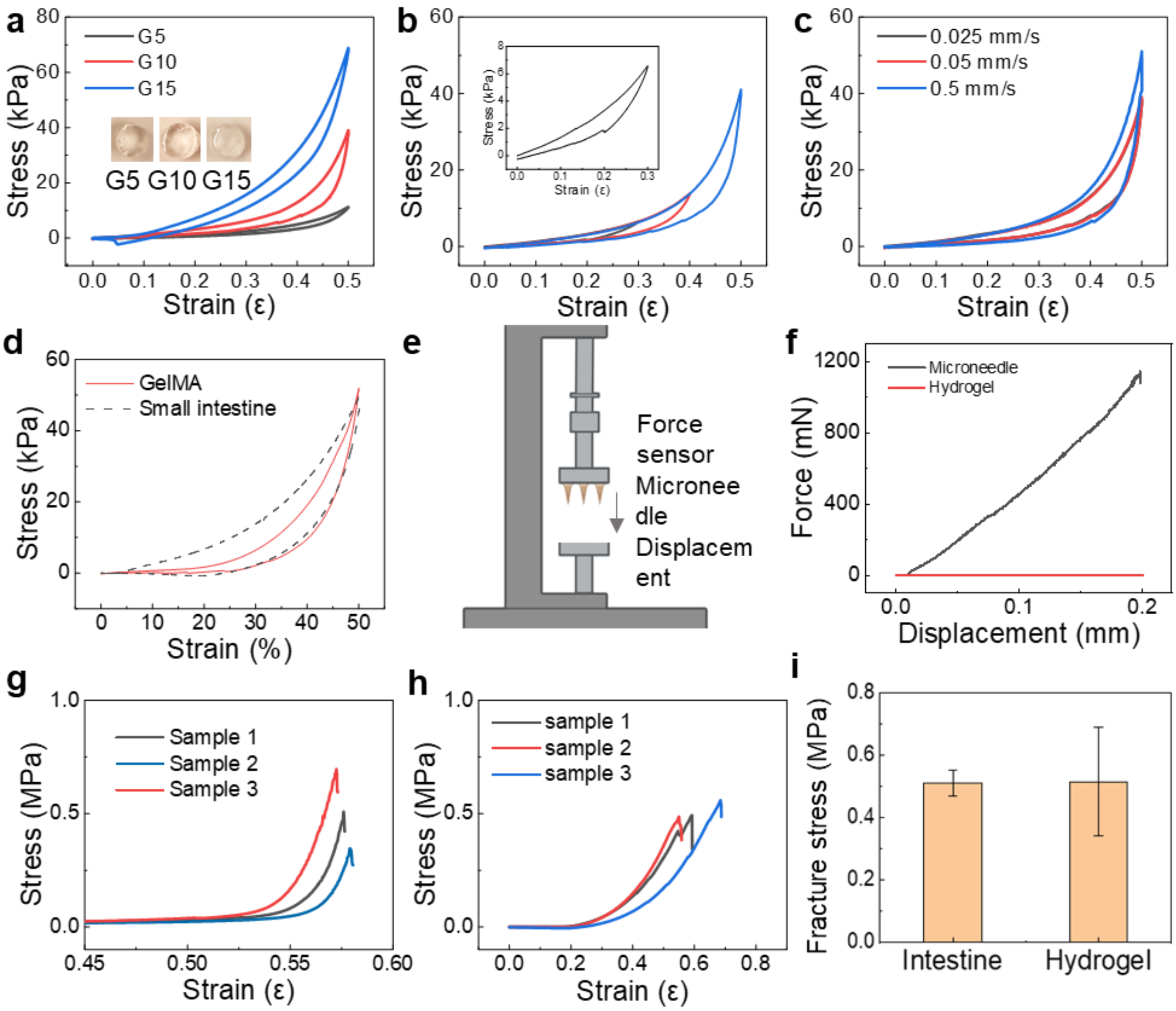

Fig. S12 Mechanical test of hydrogel tissue models. (**a**) Viscoelasticity of hydrogels with different GelMA contents (G5, G10, and G15 are 5%, 10%, and 15% GelMA). (**b**) 10% GelMA viscoelasticity versus compressive strain (30~50%). (**c**) Stress-strain relationships of hydrogels at different compression rates. (**e~f**) Mechanical test of microneedle and hydrogel tissue model, where the microneedle exhibits higher stiffness. (**g~i**) Compression fracture testing of hydrogel models and small intestine, their fracture strength are ~0.52 MPa and 0.51 MPa, respectively. Error bars represent the standard deviation (n = 3).

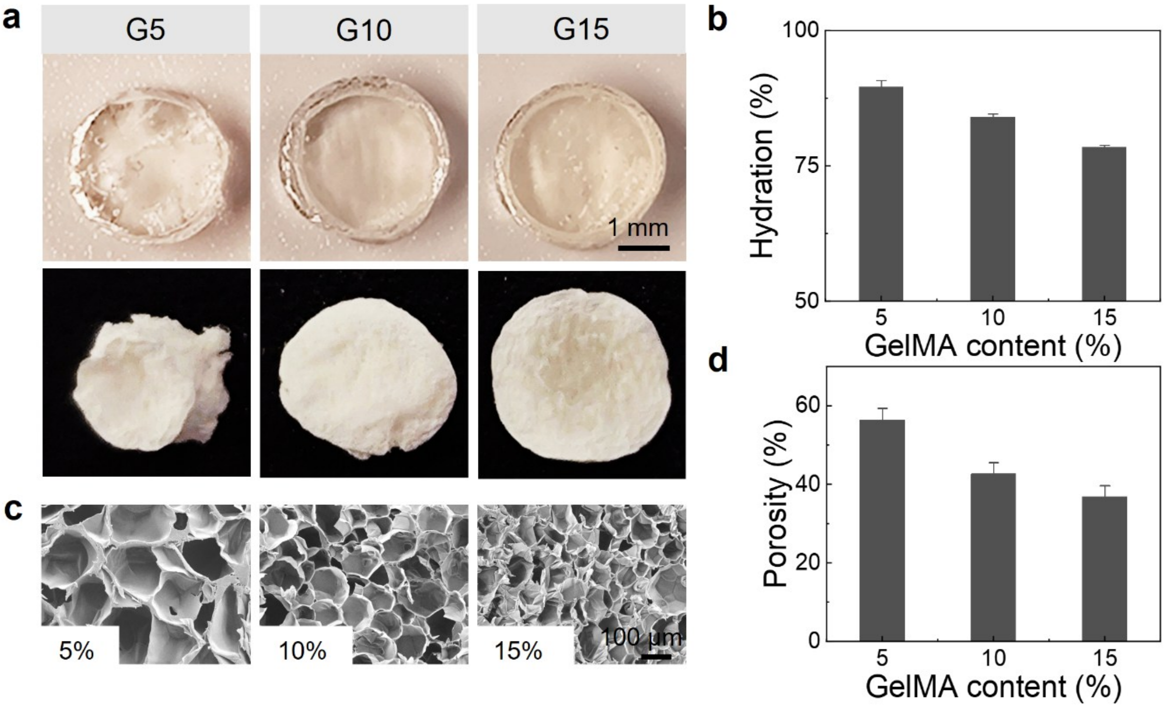

Fig. S13 Characterization of hydration levels and porosity of hydrogel tissue models. (**a**) Optical images of hydrogel models with different GelMA contents (5%, 10%, and 15%) before and after freeze-drying. (**b**) Hydration levels of hydrogel models decrease with the increase of GelMA content. All error bars are standard deviations (n=3). (**c**) SEM of micrometer pores in hydrogel models with different GelMA concentrations. (**d**) Porosity of the hydrogel model decreases with GelMA content increase. All error bars are standard deviations (n=3).

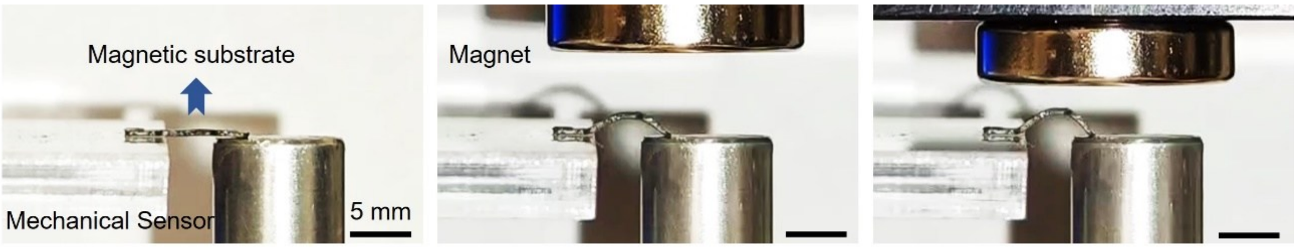

Fig. S14 The measurement process of the magnetic force applied to the USMS. One side of the magnetic substrate is placed flat on the precise mechanical sensor, and the permanent magnet is gradually approached on the other side to measure the magnetic force.

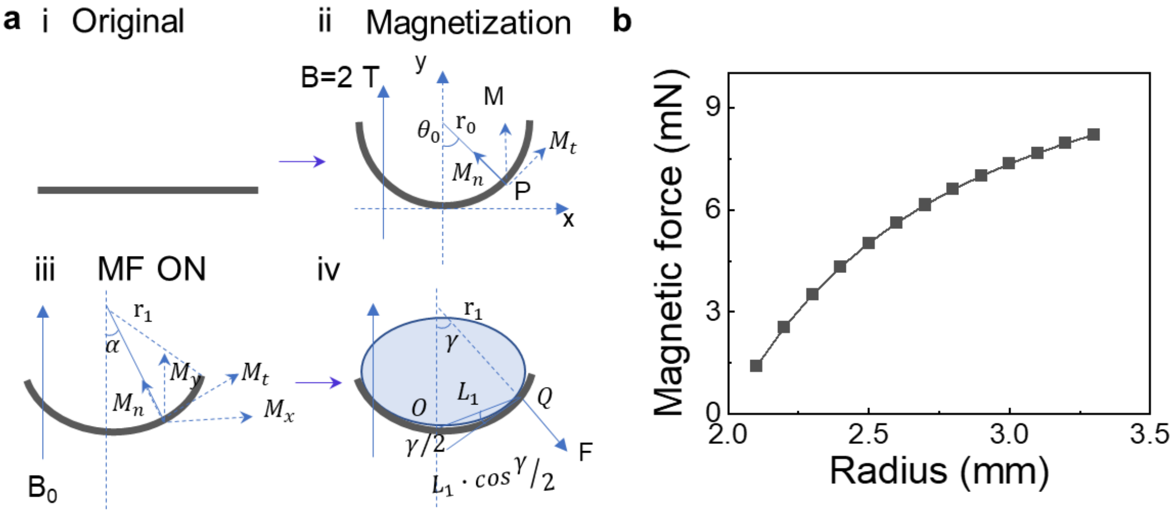

Fig. S15 (**a**) Force analysis of magnetic substrates. (**b**) As the diameter of the wrapped object increases, the penetration force provided by the robot increases.

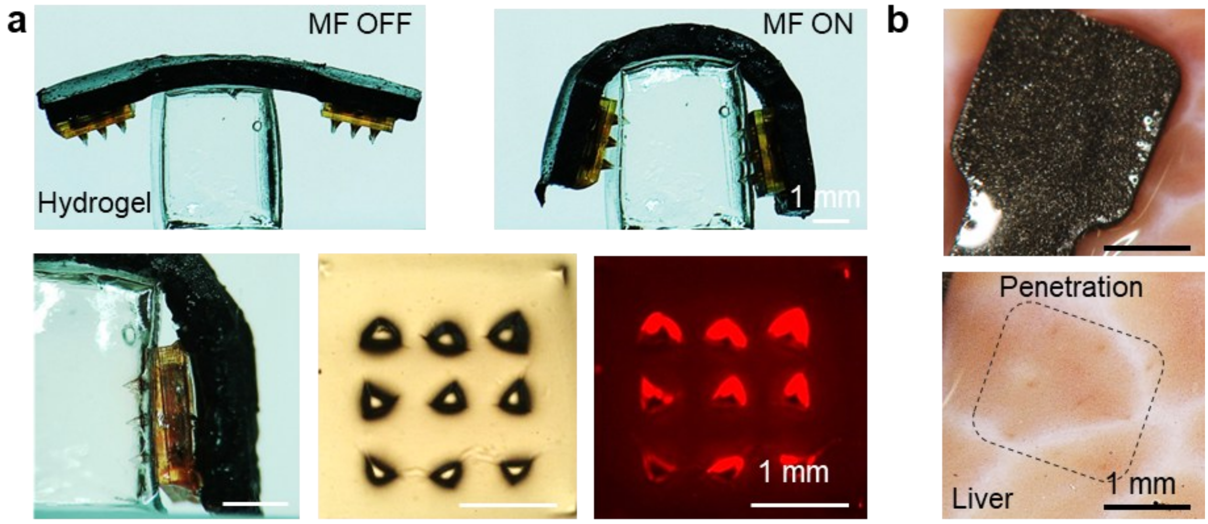

Fig. S16 (**a**) An USMS with two patches for artificial hydrogel penetration by magnetic torque. MF represents a magnetic field. (**b**) USMS penetrates the fresh pork liver. MN represents microneedle.

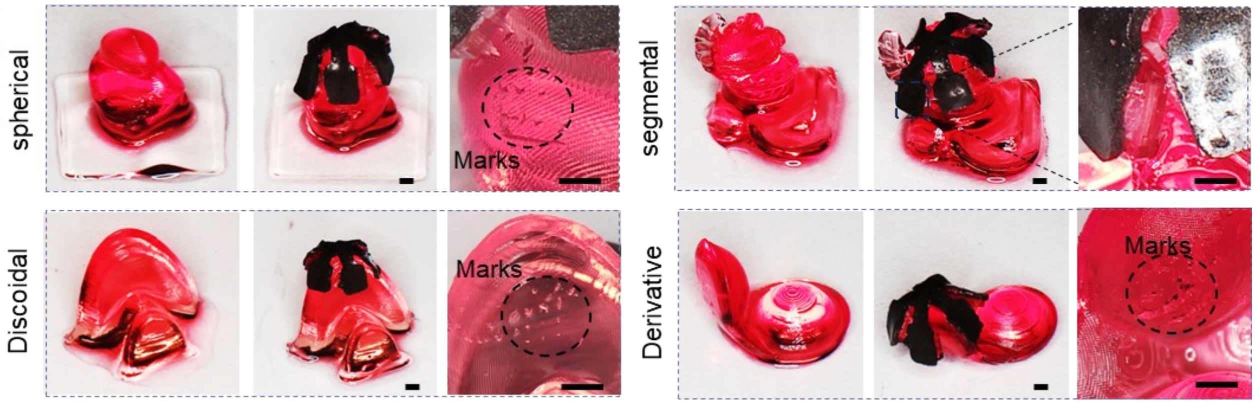

Fig. S17 Penetration characterizations of the small intestine and semi-spherical irregular hydrogel models (spherical, discoidal, segmental, and derivative mass) by the USMS. Scale bars: 1 mm.

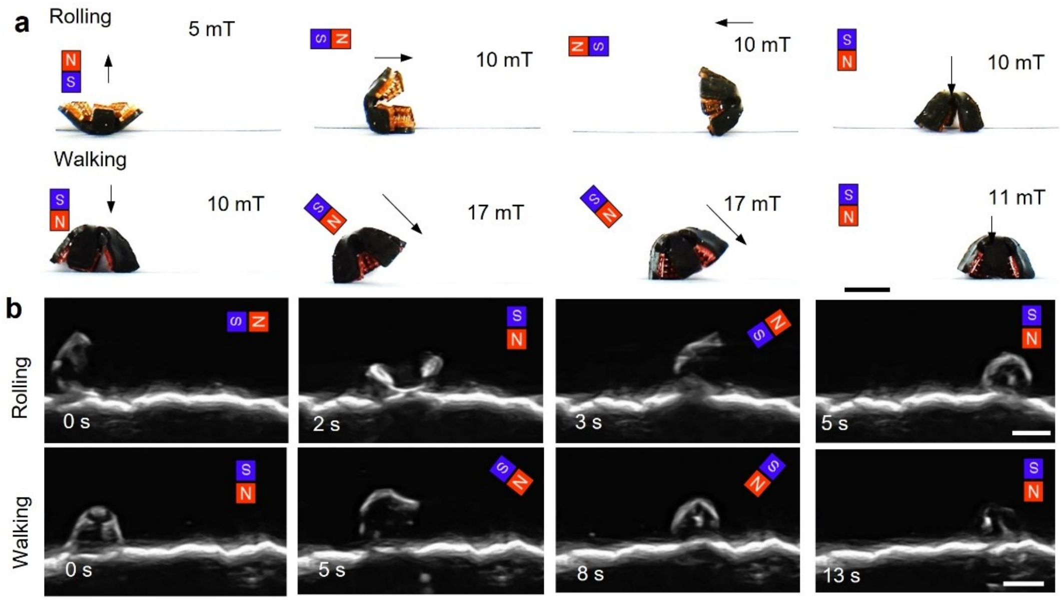

Fig. S18 (a) The rolling and walking locomotion modes of the USMS on a dry polymer surface under different dynamic magnetic fields. (**b**) The corresponding locomotion of USMS under ultrasonic navigation. All scale bars: 5 mm.

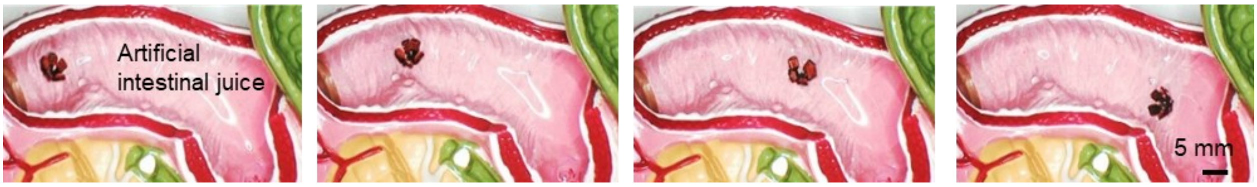

Fig. S19 The USMS rolls on the surface of a mucus-filled small intestine model.

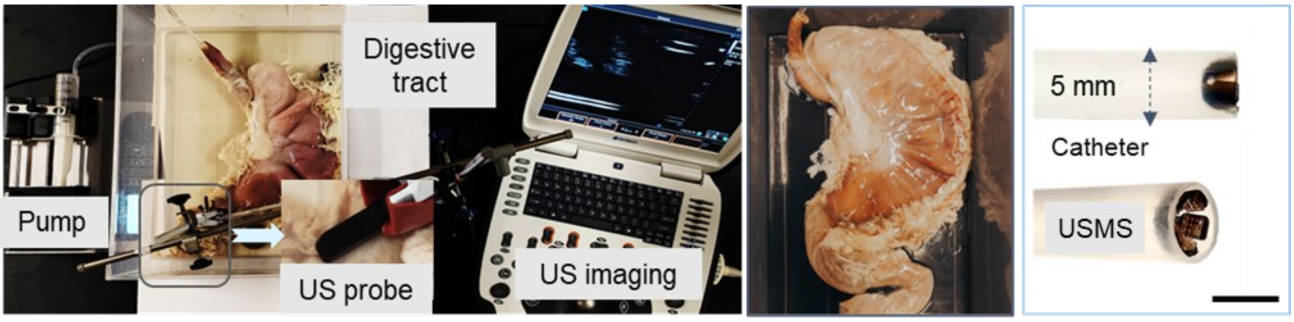

Fig. S20 The ultrasound imaging system setup consists of a pump, ultrasound probe, and image capture computer, which is used to navigate the USMS in an isolated digestive tract. Scale bars: 5 mm.

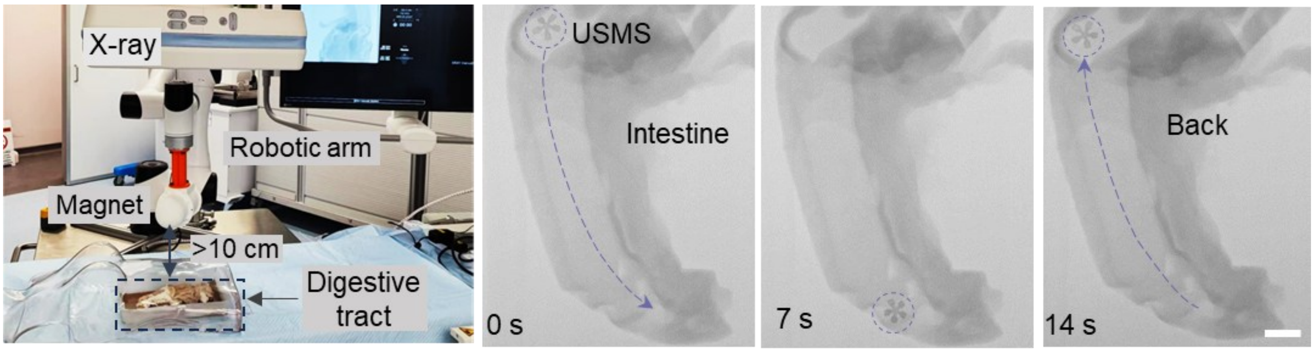

Fig. S21 The USMS rolls in the small intestine under X-ray navigation, where permanent magnets can drive USMS at large working distances (>10 cm). Scale bar, 10 mm.

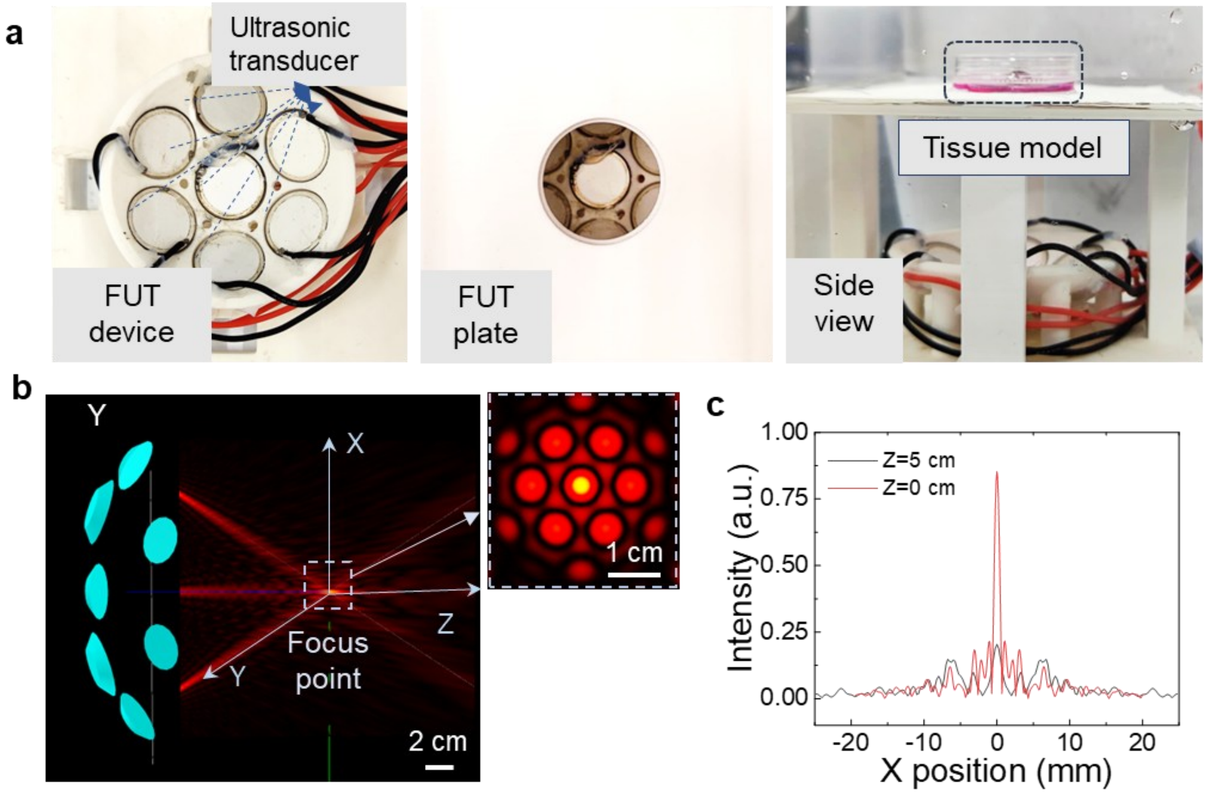

Fig. S22 (**a**) The focused ultrasound triggering (FUT) device is composed of seven ultrasonic transducers. The artificial hydrogel model filled with cells is placed in the focal area of the device for FUT. (**b**) Simulation of ultrasound distribution on XZ and XY planes. (**c**) Intensity distribution on the XY plane, where the ultrasound is focused on a spot of 2 mm diameter.

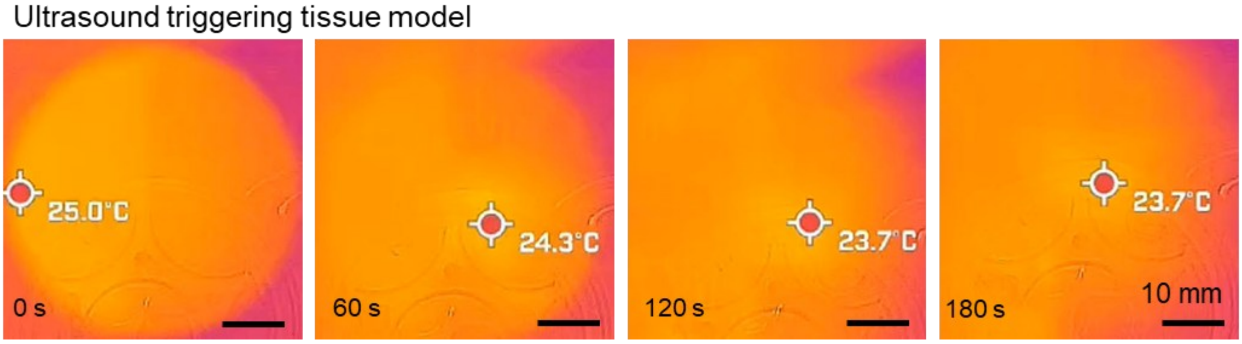

Fig. S23 In the absence of the USMS, the temperature of the hydrogel model heated with ultrasound (US) remains constant (~25 °C, room temperature).

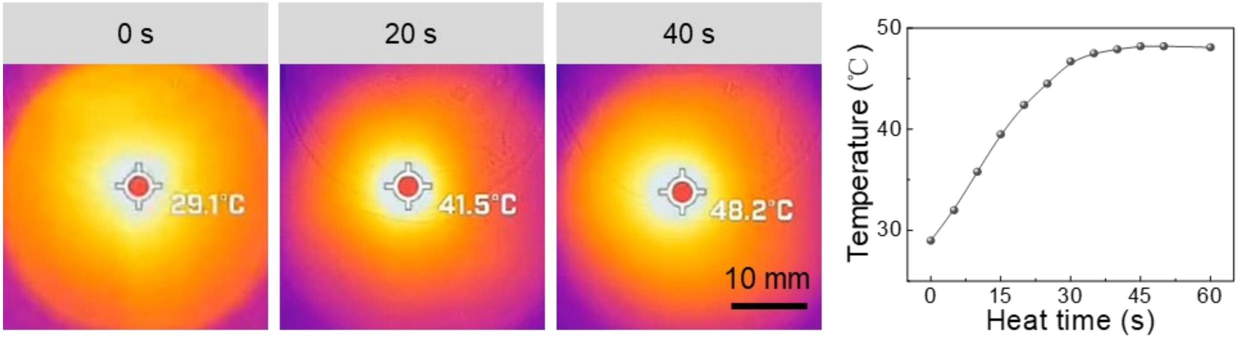

Fig. S24 Temperature of USMS under FUT within 40 s.

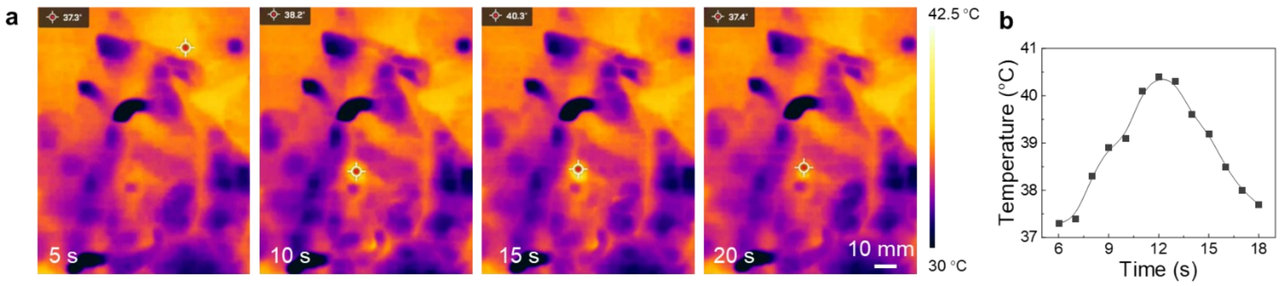

Fig. S25 Local temperature mapping in a mouse model. (**a**) Infrared thermal imaging is performed to monitor temperature changes during focused ultrasound-triggering actuation over 20 s. (**b**) The tissue temperature increased gradually with prolonged ultrasound exposure and reached a maximum of 40.3 °C at 15 s, remaining below the threshold associated with thermal injury.

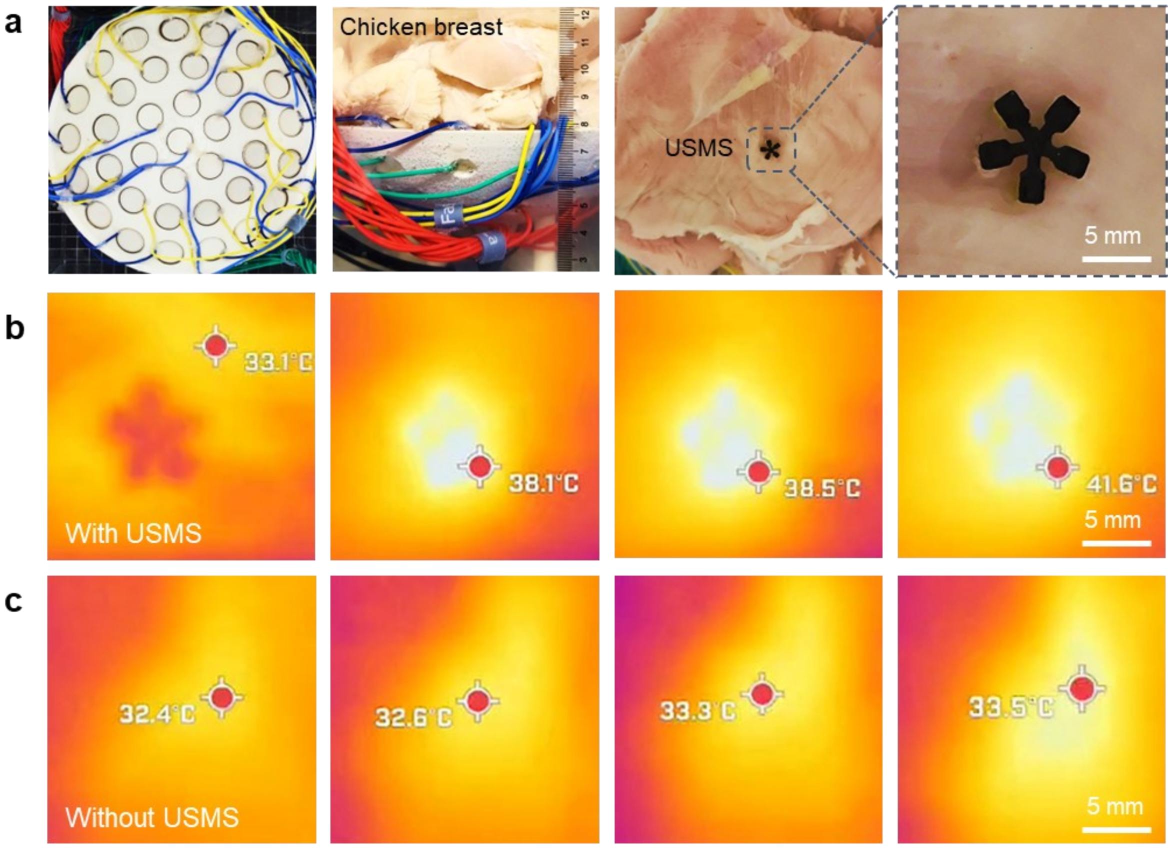

Fig. S26 Focused ultrasound heating system with long working distance for large animal experiments. (**a**) Focused ultrasound heating system with 35 ultrasound transducers. 8~10 cm thick chicken breasts were placed on the system to verify their suitability for large animal models. (**b**) Focused ultrasound can heat USMS across a ~10 cm chicken breast for active drug release. (**c**) Focused ultrasound is difficult to heat biological tissue, showing its biosafety.

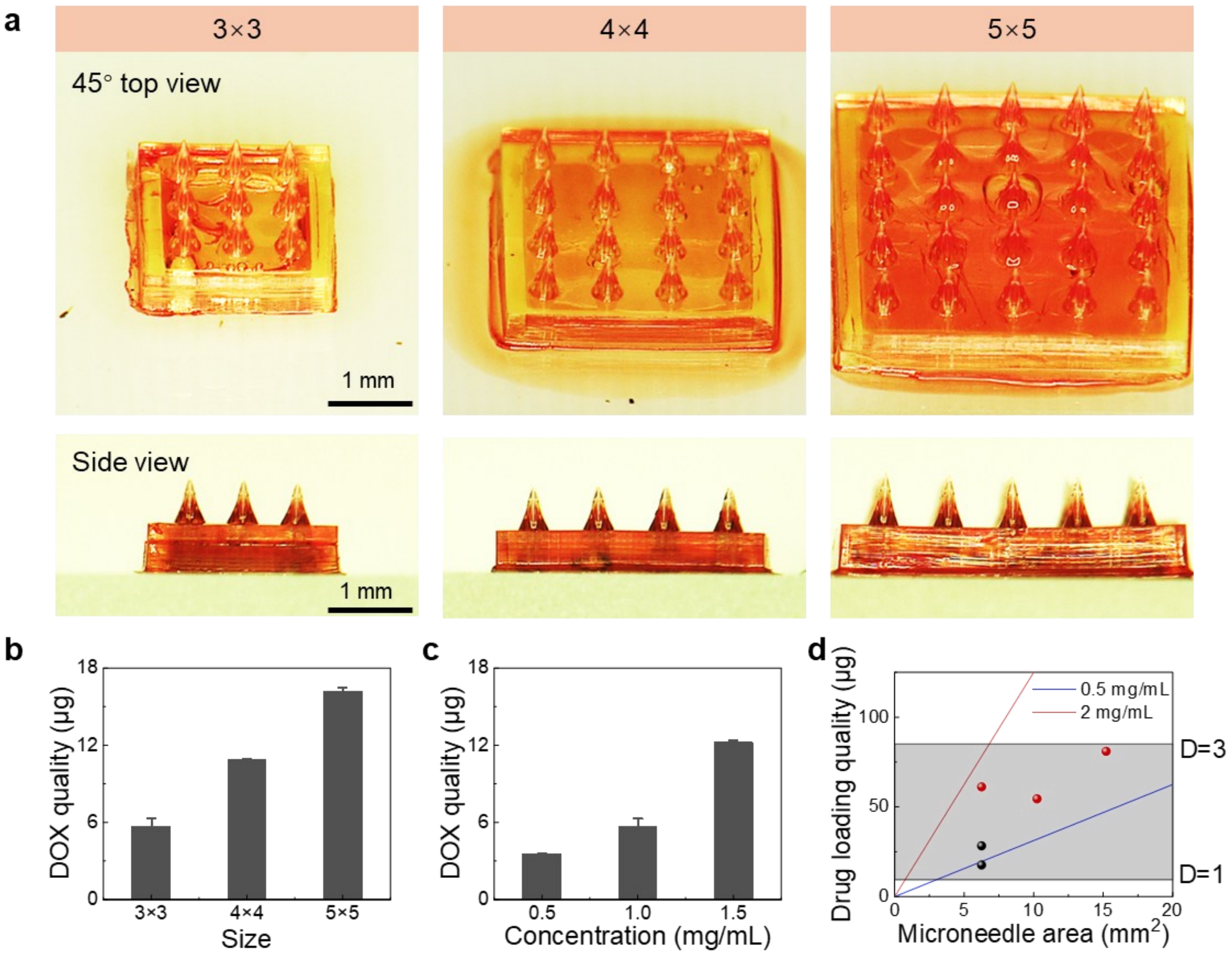

Fig. S27 Drug loading capability of microneedles. (**a**) Optical images of microneedle devices with different needle numbers after loading the drug. (**b**) Quality of the drug that can be loaded in devices with different numbers of microneedles (concentration 1 mg/mL). (**c**) Quality of the drug that can be loaded in devices with different drug concentrations (size=3×3). (**d**) Quality of chemotherapeutic drugs (DOX) required to treat tumors of different diameters (D) from 1 cm to 3 cm. All error bars are standard deviations (n=3).

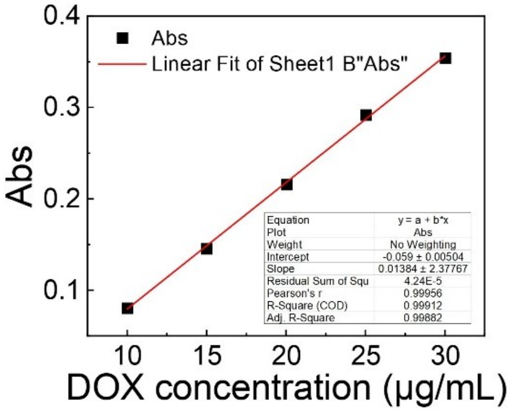

Fig. S28 Ultraviolet (UV) fluorescence spectrometry to calibrate the concentration of doxorubicin (DOX) for calculating the release of DOX.

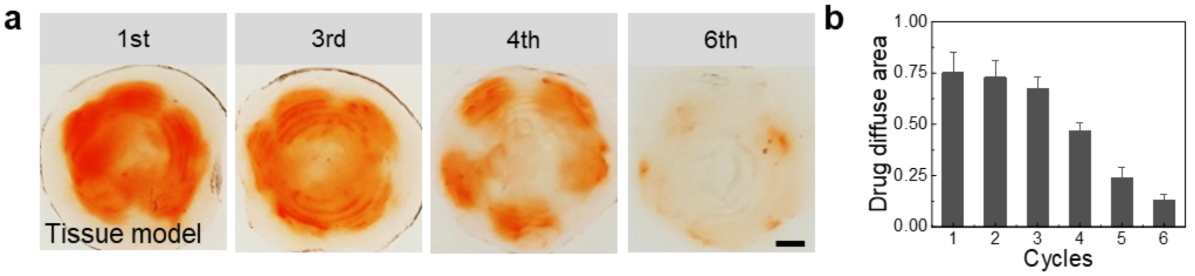

Fig. S29 USMS releases drugs multiple times in a hydrogel tissue model by ultrasound triggering, with ~75% of the drug delivery area for the 1st time and ~10% of the drug delivery area for the 6th time. Error bars represent the standard deviation (n = 3). Scale bar, 1 mm.

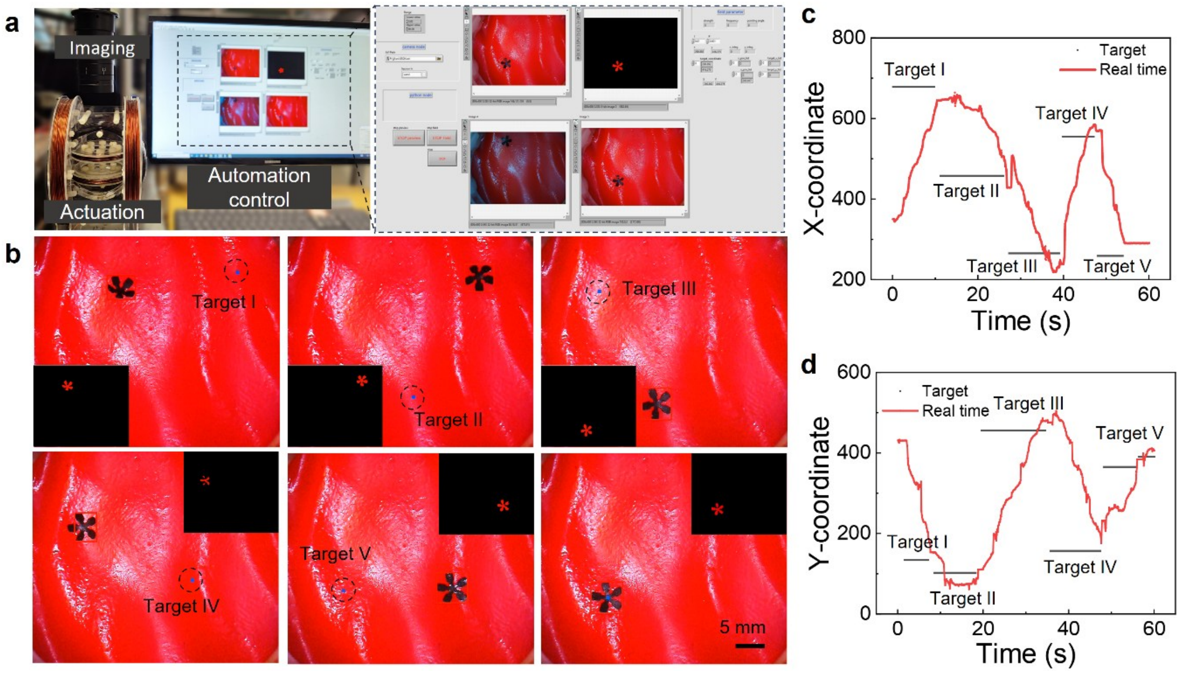

Fig. S30 USMS automatic targeting system. (**a**) The setup of an automatic targeting system mainly consists of an image capture system, a magnetic actuation system, and a computational control system. (**b**) USMS automatically targets five locations. (**c**~**d**) A comparison of the XY coordinates of the USMS's target and real-time positions demonstrates the precision and stability of its automatic position adjustment.

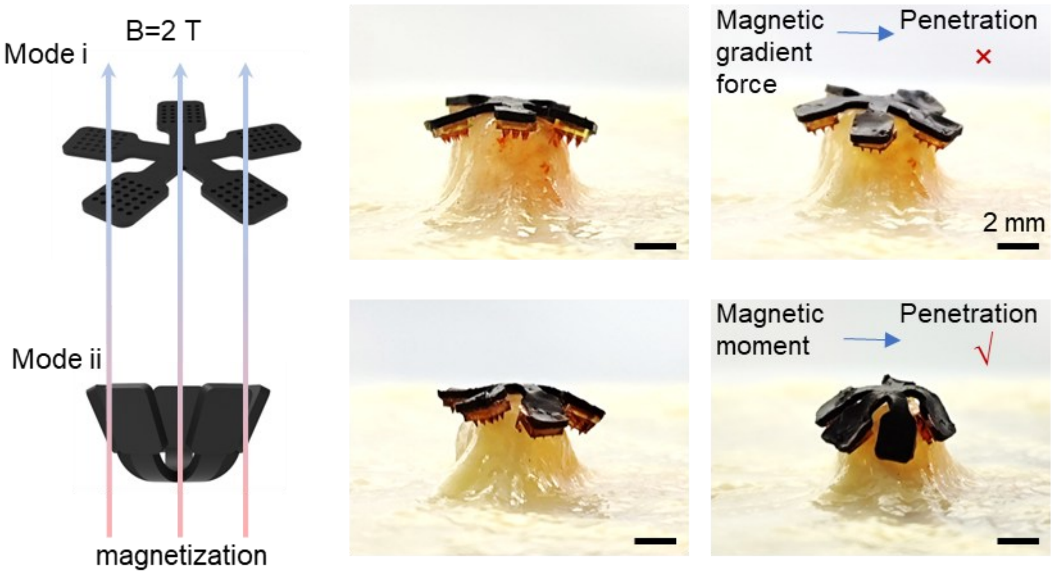

Fig. S31 The USMS with different magnetization modes, where the USMS (ii) demonstrates wrap-around penetration of 3D target tissue and the USMS (i) exhibits no penetration.

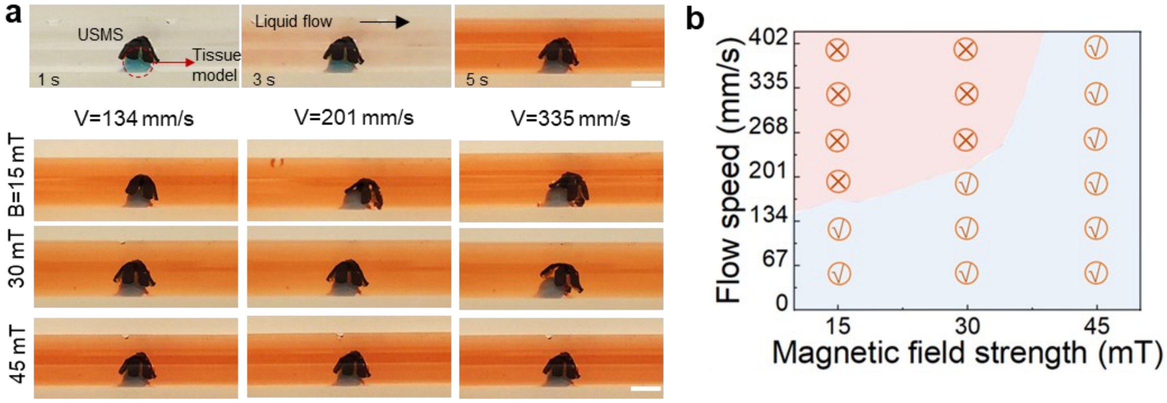

Fig. S32 USMS resists fluid flow. (**a**) The USMS wraps and penetrates the target tissue model under liquid flow (335 mm/s) under 45 mT magnetic strength. (**b**) The higher the strength of the magnetic field strength, the stronger the USMS against liquid flow. All scale bars: 5 mm.

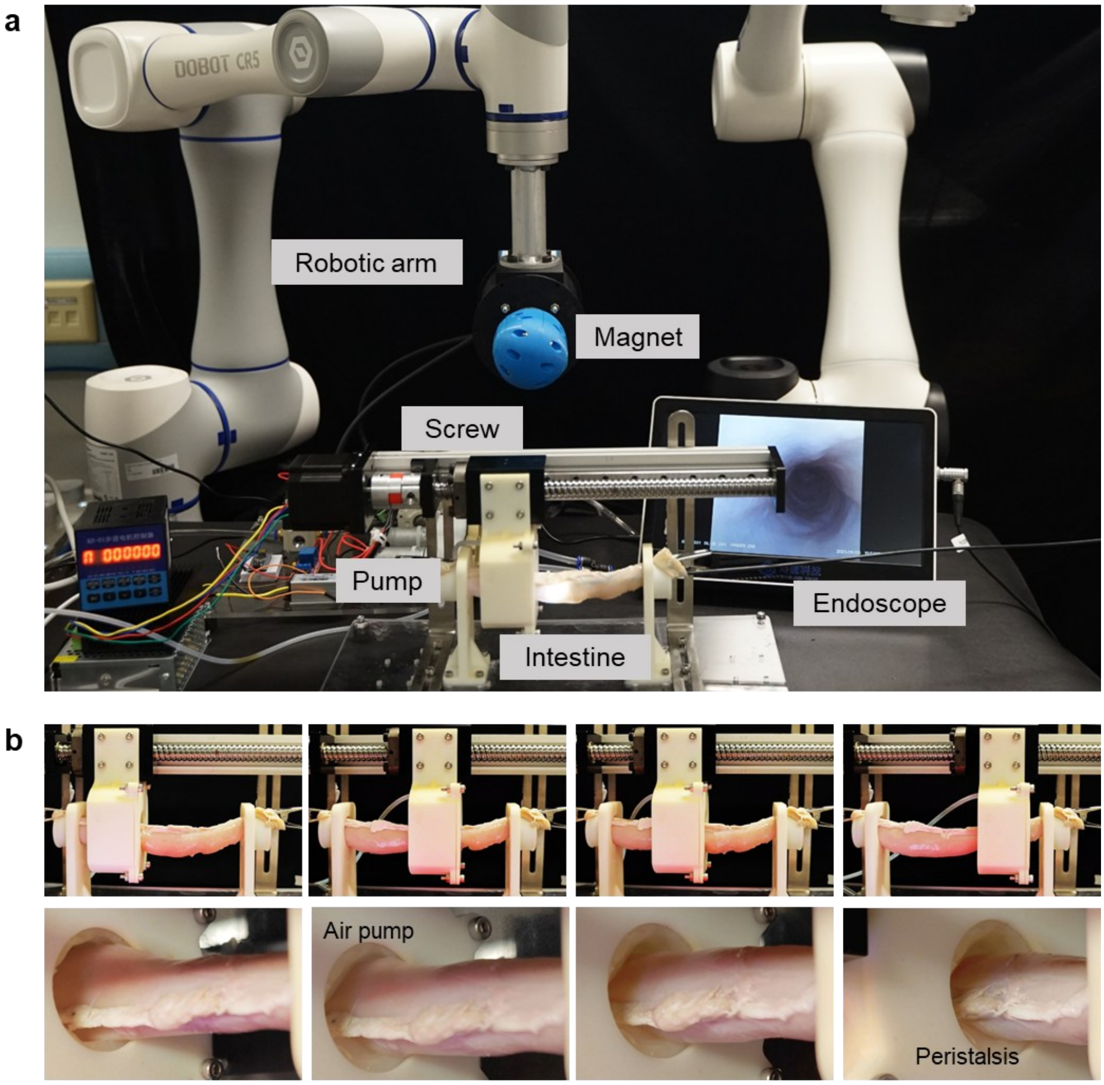

Fig. S33 (**a**) Intestinal peristalsis test setup built with a removable air pump to simulate compression and shear forces generated by smooth muscle. (**b**) Intestinal peristalsis under air pump compression and shear.

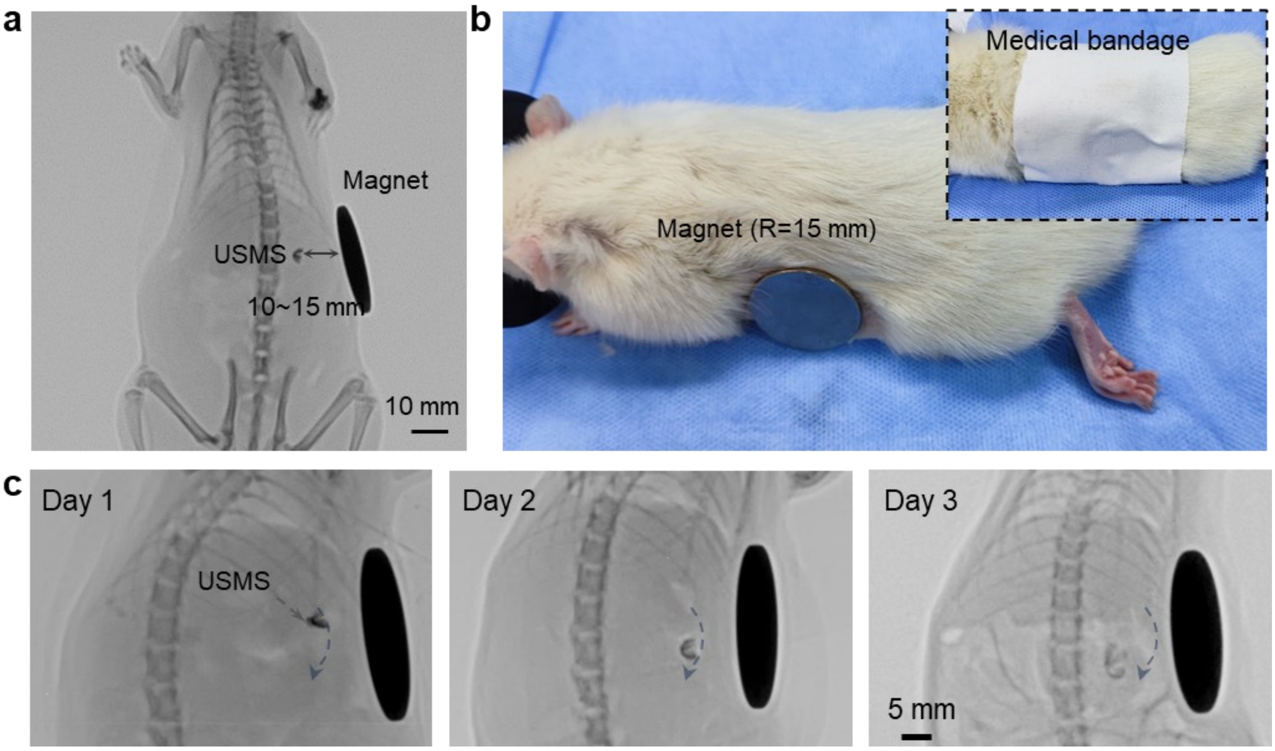

Fig. S34 Characterization of USMS against shear forces. (**a**) Images of the magnet's position relative to the rat's stomach. (**b**) Optical time-lapse image of a USMS fighting shear only in a magnetic field. (**c**) Position stability of the USMS in the rat's stomach is only under magnetic influence.

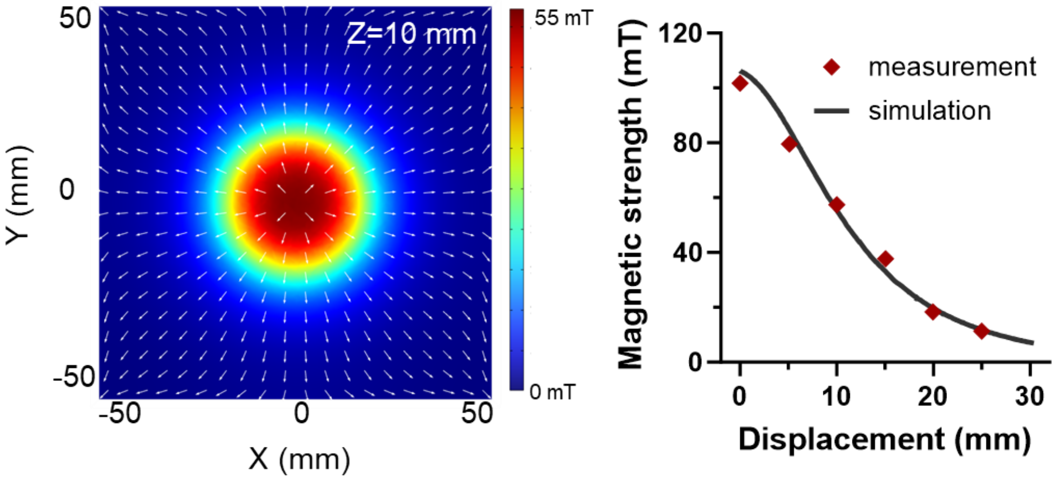

Fig. S35 Simulation of magnetic field strength at a plane 10 mm away from the magnet and quantitative relationship between magnetic field strength and distance.

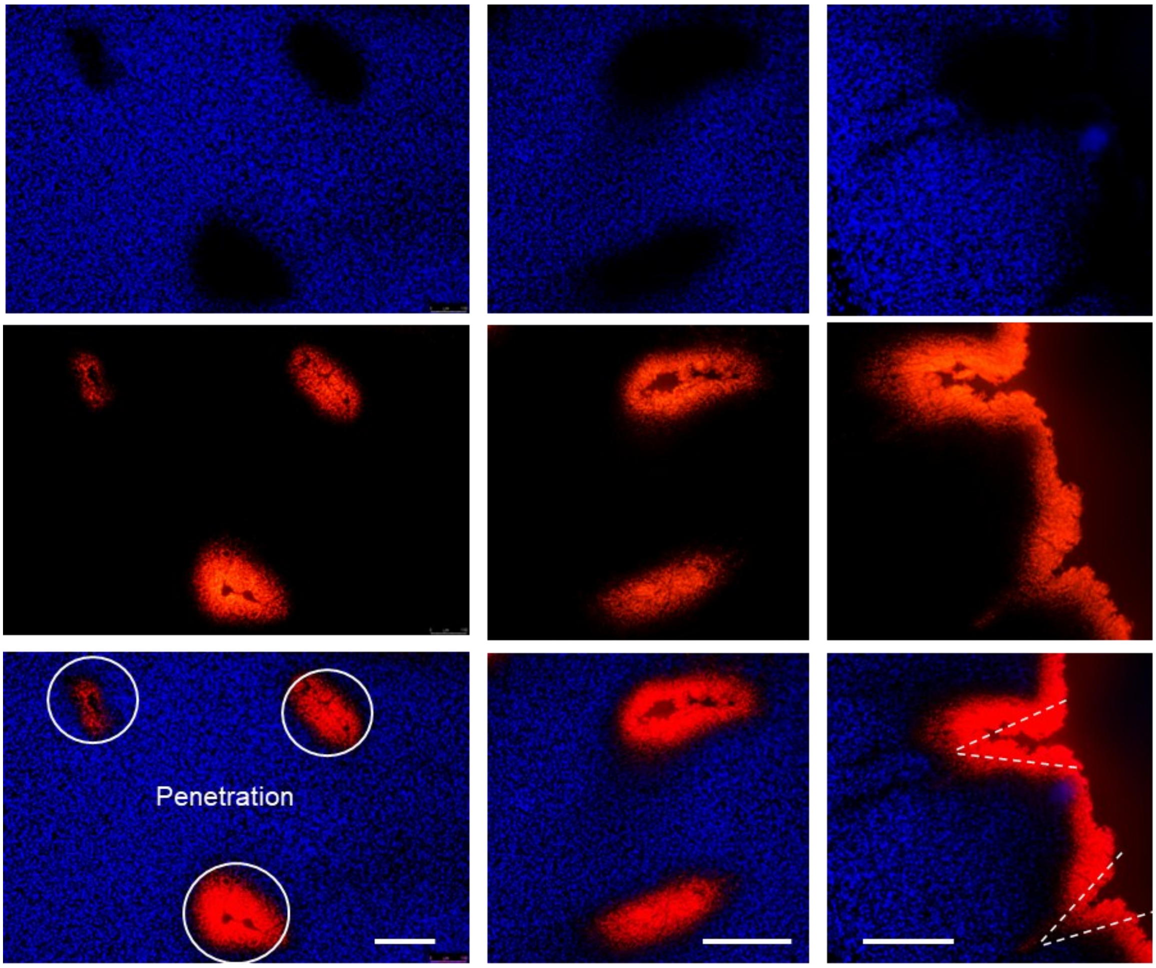

Fig. S36 Fluorescence micrographs of frozen sections of tissues from the mouse stomach after USMS penetration for on-demand drug delivery. All scale bars: 300 μm.

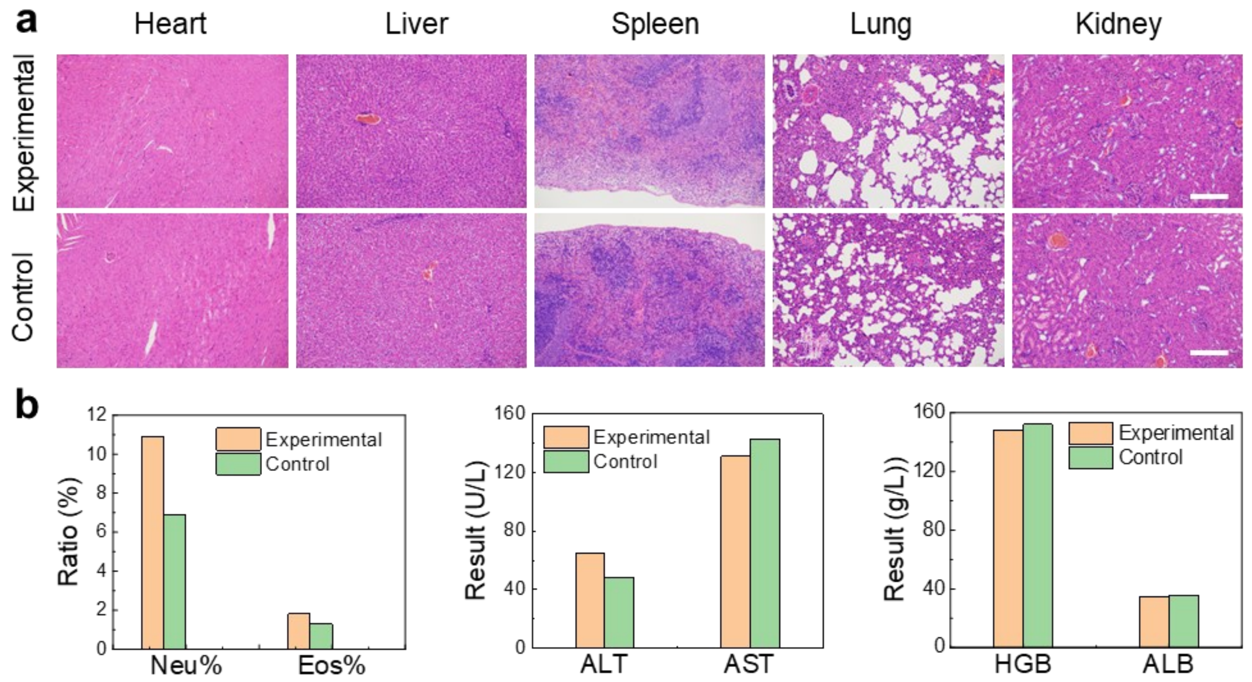

Fig. S37 Toxicity evaluation of USMS. (**a**) Sections of the mouse heart, liver, spleen, lung, and kidney were shown by H&E staining. (**b**) The biochemical test results of the experimental and control groups after the anchoring test showed no significant difference in all indicators, which verified the biosafety of USMS. All scale bars: 100 μm.

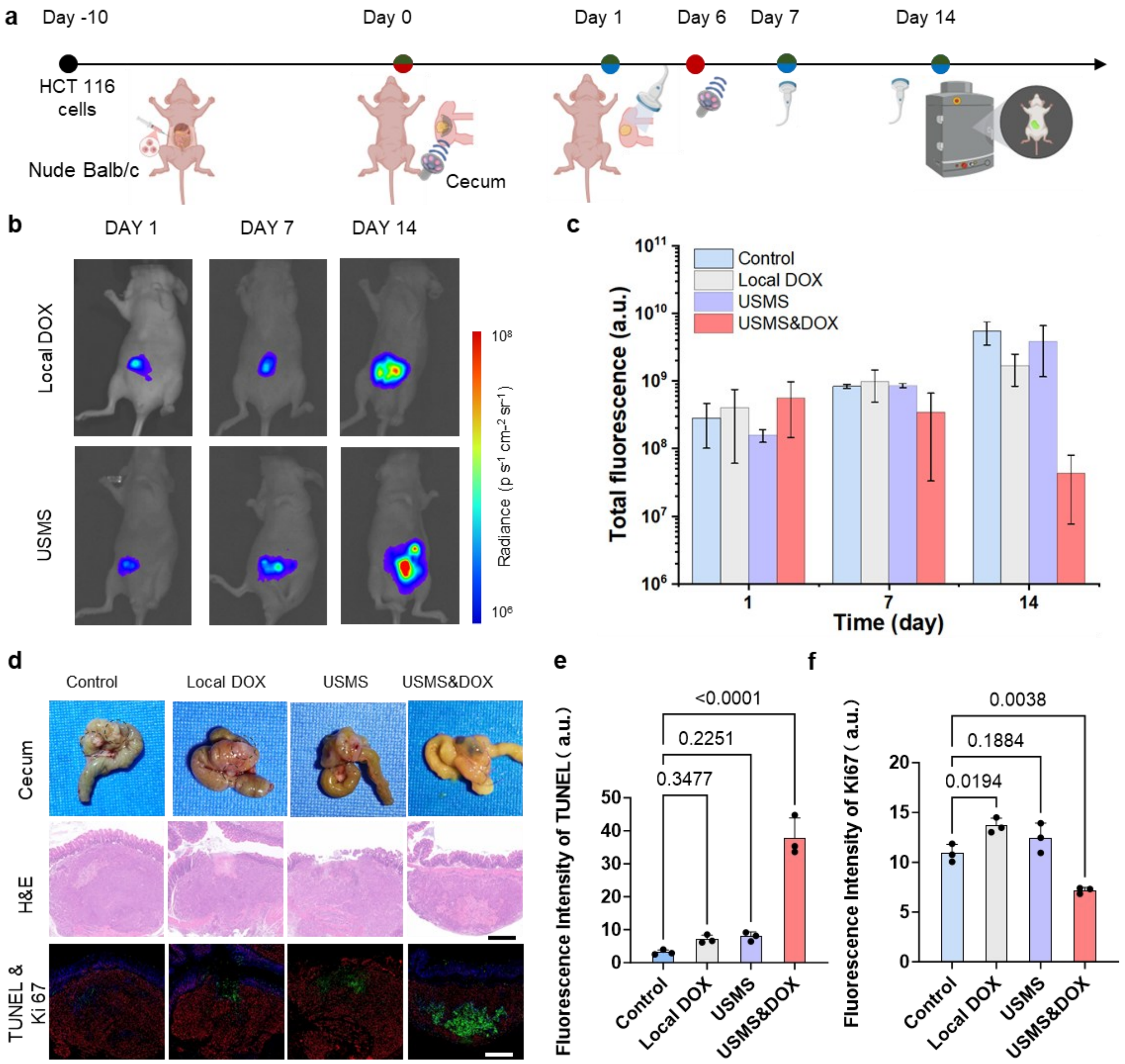

Fig. S38 In vivo validation of the on-demand drug delivery, anchoring, and sensing capabilities of USMS in an orthotopic colorectal tumor model. (**a**) Schematic illustration of the orthotopic colorectal tumor model in mice and the process of USMS-mediated targeted delivery, and therapeutic evaluation via ultrasound and fluorescence imaging. Fluorescence and ultrasound imaging were employed for efficacy assessment. (**b**) Representative *in vivo* fluorescence images of mice (Local DOX and USMS group) at different points post-treatment. (**c**) Quantitative statistical analysis of tumor fluorescence intensity in mice during a 14-day treatment period. (**d**) Representative images of colorectal cancer tissues and histological evaluation of tumor suppression by H&E staining along with IHC detection of TUNEL and Ki67. Statistical analysis of Ki67 (**e**) and TUNEL (**f**) expression across different experimental groups. Error bars represent the standard deviation (n = 3). Scale bars: 300 µm.

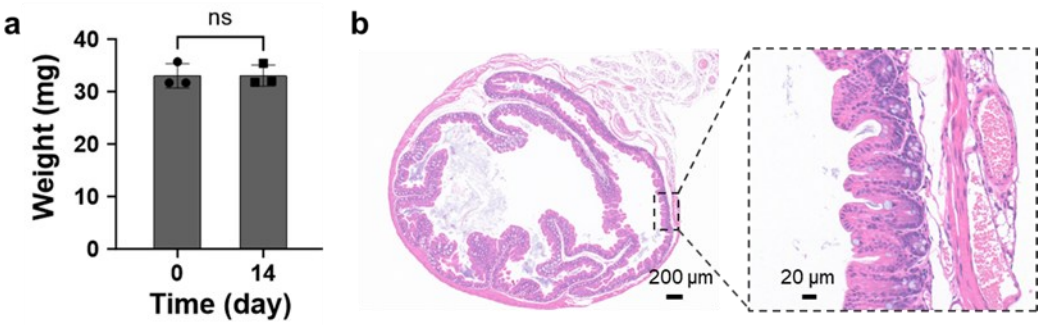

Fig. S39 Stability of the magnetic substrate after in vivo test. (**a**) Quantitative weight measurements of the magnetic substrate at Day 0 and Day 14 following immersion or in vivo retention. No significant difference was observed between time points. Error bars represent the standard deviation (n = 3). (**b**) H&E staining images of the intestines showed no leakage of NdFeB particles.

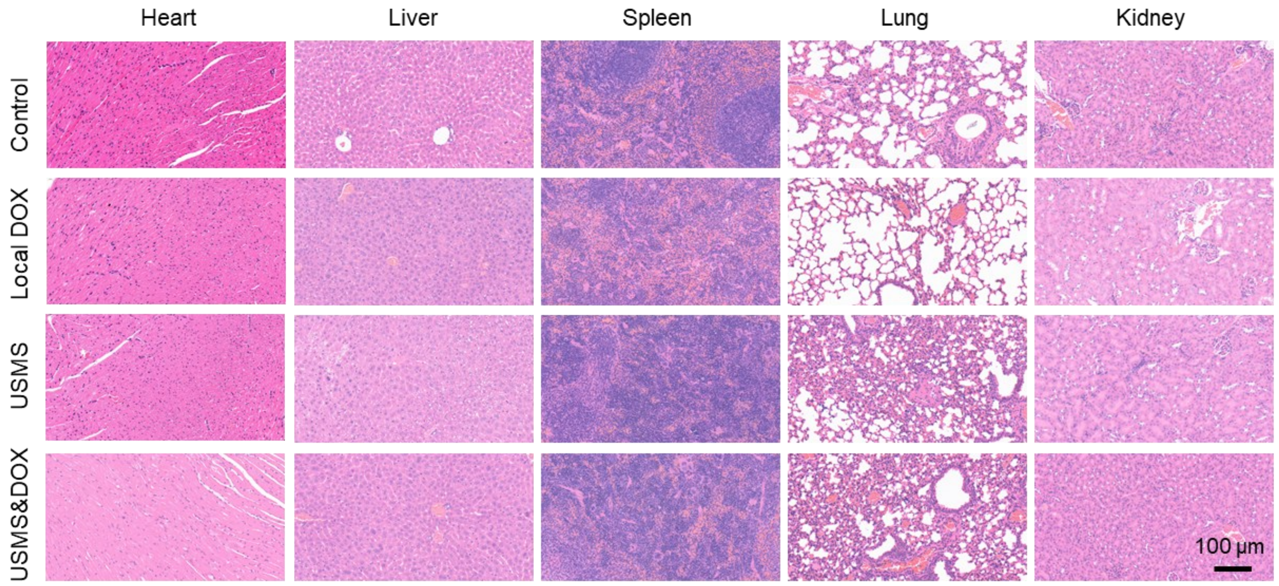

Fig. S40 H&E staining of histological sections of the mice in the control, Local DOX, USMS, and USMS&DOX groups. Liver, spleen, lung, and kidney tissues were collected at the end of the study.

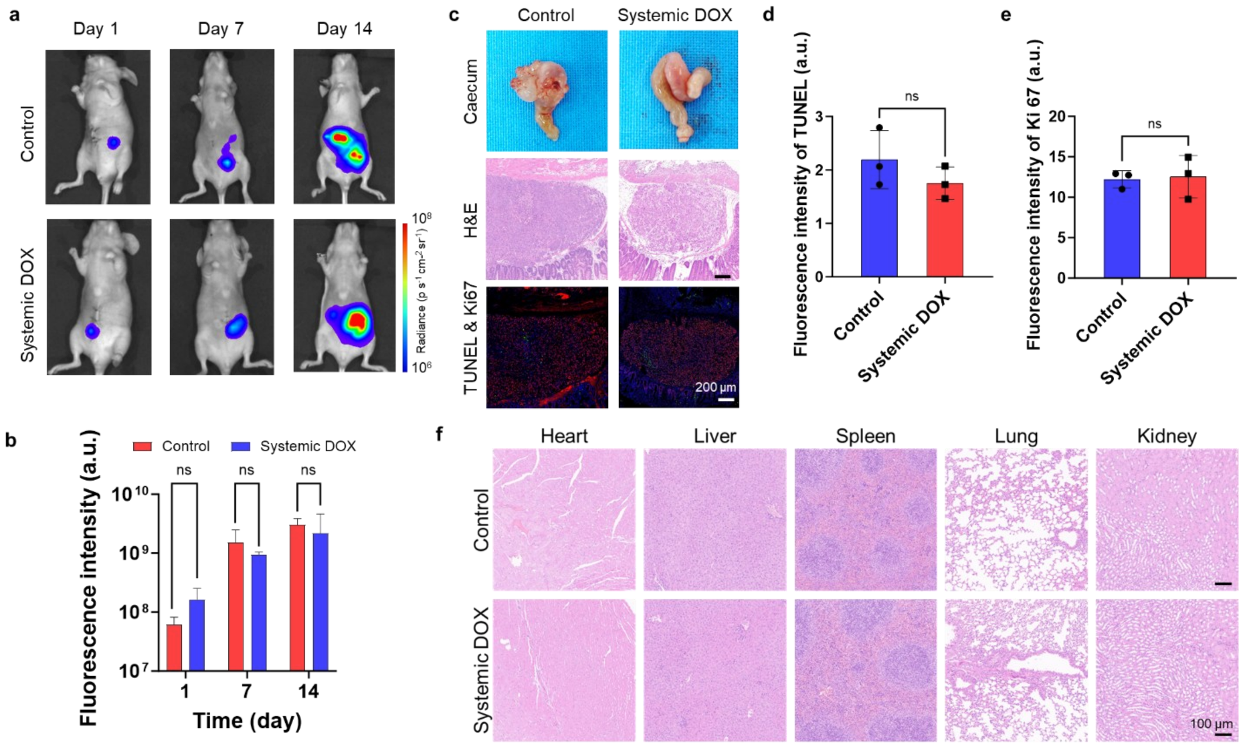

Fig. S41 In vivo tumor therapy of control and systemic DOX groups. (**a**) Representative in vivo fluorescence images of tumor-bearing mice from the control and systemic DOX groups. (**b**) Quantitative analysis of tumor fluorescence signals is shown on the right. (**c**) Representative gross images, H&E staining, and immunofluorescence analysis of colonic tumor tissues from control and systemic DOX groups. H&E staining revealed comparable tumor architecture and tumor burden between groups. Immunofluorescence analysis showed no evident reduction in tumor-associated signals following systemic DOX treatment. (**d~e**) Quantitative analysis of TUNEL and Ki 67 demonstrated no significant therapeutic improvement compared with control. (**f**) Representative H&E-stained sections of heart, liver, spleen, lung, and kidney tissues collected from control and systemic DOX groups. No evident histopathological abnormalities, inflammatory infiltration, or structural damage were observed in the systemic DOX group compared with the control group. Error bars represent the standard deviation (n = 3).

References

1. Lum GZ, Ye Z, Dong X *et al.* Shape-programmable magnetic soft matter. *Proc Natl Acad Sci USA* 2016; **113**: E6007-15.
2. Gao X, Li J, Li J *et al.* Pain-free oral delivery of biologic drugs using intestinal peristalsis–actuated microneedle robots. *Sci Adv* 2024; **10**: eadj7067.
3. Ghosh A, Li L, Xu L *et al.* Gastrointestinal-resident, shape-changing microdevices extend drug release in vivo. *Sci Adv* 2020; **6**: eabb4133.
4. Abramson A, Caffarel-Salvador E, Khang M *et al.* An ingestible self-orienting system for oral delivery of macromolecules. *Science* 2019; **363**: 611-5.
5. Abramson A, Caffarel-Salvador E, Soares V *et al.* A luminal unfolding microneedle injector for oral delivery of macromolecules. *Nat Med* 2019; **25**: 1512-18.
6. Zhang Y, Wang Q, Yi S *et al.* 4D Printing of Magnetoactive Soft Materials for On-Demand Magnetic Actuation Transformation. *ACS Appl Mater Interfaces* 2021; **13**: 4174-84.
7. Zheng Z, Wang H, Demir SO *et al.* Programmable aniso-electrodeposited modular hydrogel microrobots. *Sci Adv* 2022; **8**: eade6135.
8. Zhang F, Li Z, Duan Y *et al.* Gastrointestinal tract drug delivery using algae motors embedded in a degradable capsule. *Sci Robot* 2022; **7**: eabo4160.
9. Zhang X, Chen G, Fu X *et al.* Magneto-Responsive Microneedle Robots for Intestinal Macromolecule Delivery. *Adv Mater* 2021; **33**: e2104932.

Supplementary Movie S1.
Penetration of small intestine and hydrogel tissue model by the microneedle.

Supplementary Movie S2.
USMS penetrates hydrogel tissue model by magnetic force.

Supplementary Movie S3.
Targeted drug delivery and automatic position adjustments of the USMS.

Supplementary Movie S4.
Targeted locomotion of USMS guided by medical imaging.

Supplementary Movie S5.
Testing of USMS against shear force.

Supplementary Movie S6.
Verification of USMS's strong locomotion and anchoring capabilities.
